# Cytokinin Plant Hormones Have Neuroprotective Activity in In Vitro Models of Parkinson’s Disease

**DOI:** 10.3390/molecules26020361

**Published:** 2021-01-12

**Authors:** Gabriel Gonzalez, Jiří Grúz, Cosimo Walter D’Acunto, Petr Kaňovský, Miroslav Strnad

**Affiliations:** 1 Laboratory of Growth Regulators, Institute of Experimental Botany of the Czech Academy of Sciences, and Faculty of Science, Palacký University, Šlechtitelů 27, CZ-78371 Olomouc, Czech Republic; Gonzalez.gabriel@seznam.cz (G.G.); jiri.gruz@upol.cz (J.G.); waldacun@gmail.com (C.W.D.); 2 Department of Neurology, University Hospital Olomouc and Faculty of Medicine and Dentistry, Palacký University Olomouc, CZ-775 20 Olomouc, Czech Republic; Petr.Kanovsky@fnol.cz

**Keywords:** cytokinin, phytohormone, neuroprotection, neuron-like SH-SY5Y cells, cytotoxicity, salsolinol, glutamate, oxidative stress, Parkinson’s disease

## Abstract

Cytokinins are adenine-based phytohormones that regulate key processes in plants, such as cell division and differentiation, root and shoot growth, apical dominance, branching, and seed germination. In preliminary studies, they have also shown protective activities against human neurodegenerative diseases. To extend knowledge of the protection (protective activity) they offer, we investigated activities of natural cytokinins against salsolinol (SAL)-induced toxicity (a Parkinson’s disease model) and glutamate (Glu)-induced death of neuron-like dopaminergic SH-SY5Y cells. We found that kinetin-3-glucoside, *cis*-zeatin riboside, and N^6^-isopentenyladenosine were active in the SAL-induced PD model. In addition, *trans*-, *cis*-zeatin, and kinetin along with the iron chelator deferoxamine (DFO) and the necroptosis inhibitor necrostatin 1 (NEC-1) significantly reduced cell death rates in the Glu-induced model. Lactate dehydrogenase assays revealed that the cytokinins provided lower neuroprotective activity than DFO and NEC-1. Moreover, they reduced apoptotic caspase-3/7 activities less strongly than DFO. However, the cytokinins had very similar effects to DFO and NEC-1 on superoxide radical production. Overall, they showed protective activity in the SAL-induced model of parkinsonian neuronal cell death and Glu-induced model of oxidative damage mainly by reduction of oxidative stress.

## 1. Introduction

Parkinson’s disease (PD) is the second most common motor-related neurodegenerative disease, and numbers of globally diagnosed cases are expected to rise from 6 million in 2015 to more than 12 million by 2040 [1]. It is characterized by motor symptoms linked with specific degeneration and loss of approximately 30–70% of dopaminergic (DA) neurons in the substantia nigra pars compacta and their projections to the striatum [2,3]. Some, of many, known molecular hallmarks of PD include enhanced oxidative and nitrosative stress, mitochondrial dysfunction [4,5,6,7], excitotoxicity [8], ubiquitin/proteasomal system dysfunction [9], and neuroinflammation [10]. Current treatments have various adverse side-effects and only offer symptomatic relief [11], so there are intense efforts to develop drugs with efficient curative effects on degenerating DA neurons. Resources that may aid such efforts include natural compounds that tend to have fewer side effects. Inter alia, substances from *Ginkgo biloba* (ginkgetin, ginkgolide, bilobalide), ginseng (ginsenosides), and flavonoids (baicalein, kaempferol, rutin and luteolin) have demonstrated broad protective activity in several in vitro models (including the human neuroblastoma cell line SH-SY5Y) and in vivo models of PD induced by 1,1’-dimethyl-4,4’-bipyridinium dichloride (paraquat), 1-methyl-4-phenyl-1,2,3,6-tetrahydropyridine (MPTP), 1-methyl-4-phenylpyridinium (MPP+), and 6-hydroxydopamine (6-OHDA) [12].

The study presented here focused on effects of a class of natural phytohormones called cytokinins (CKs), and their metabolites, which are well known regulators of cell division, growth, differentiation, and leaf senescence in plants [13]. Structurally, CKs are adenine derivatives substituted at the N^6^-position with either a prenyl (isopentenyl) or aromatic sidechain. Natural forms include 6-(E)-4-hydroxy-3-methylbut-2-enylaminopurine (*trans*-zeatin, ***t*Z**), its 6-(Z)-isomer (*cis*-zeatin, ***c*Z**), N^6^-isopentenyladenine (**iP**), 6-benzylaminopurine (**BAP**), 6-furfurylaminopurine (kinetin, **K**), and *ortho-*, *meta*-, and *para*-hydroxylated or methoxylated derivatives of **BAP**, called topolins (***o*T**, ***m*T**, ***p*T**, **Me*o*T**, **Me*m*T**, **Me*p*T**). Various 9-ribosides, 9-nucleotides, as well as 7-, 9, and O-glucosides of these forms also commonly occur, as shown in Table 1. In addition to their native roles in plants, CKs have shown potent ant-oxidant activity towards reactive oxygen species (ROS) that provides protection in several in vitro stress models of aging-associated disorders [14].

In particular, CKs reportedly have cytoprotective activity in models such as H_2_O_2_-induced cell death of human fibroblasts [15] and D-galactose-induced glycoxidative stress in rat astrocytes [16]. More importantly, in this context, they have shown neuroprotective effects in models related to neurodegenerative diseases such as familial PD, proteasome inhibitor MG 132-induced or H_2_O_2_-induced toxicity in SH-SY5Y cells [17], Glu-induced oxidative damage of HT22 mouse hippocampal neuronal cells [18], and the PC12 cell model of Huntington’s disease [19]. Other studies indicate that CKs’ protective activities involve both direct [20,21] and indirect [15,16,22] modulation of cellular redox systems. In addition to CKs’ characteristic antioxidant activities, they reportedly have regulatory effects in mitochondria that enhance neuronal viability [17]. Moreover, **K** can stabilize mitochondrial membrane potential and increase ATP production, thereby mitigating Glu-induced death of HT22 cells [18]. However, despite findings regarding their effects in several models, there is limited knowledge of CKs’ protective activity in the most common (sporadic) form of PD.

To address the knowledge gap described above, we systematically evaluated effects of natural CKs and their metabolites in two in vitro models: A salsolinol (SAL)-induced model of PD and glutamate (Glu)-induced model of oxidative damage in neuron-like SH-SY5Y cells. This line was used because of its dopaminergic phenotype, sensitivity to dopaminergic toxins such as SAL, and convenient formation of relatively stable populations of differentiated neuronal cells with reduced proliferation rates following 48 h exposure to 10 µM *all-trans* retinoic acid (ATRA) [23,24,25].

Neuron-like cells were exposed to the endo/exotoxin SAL to mimic PD pathology via dysfunction of cellular redox system: Depletion of the glutathione (GSH), and inhibition of both anti-oxidant enzyme (Cu/Zn superoxide dismutase and catalase) activities and mitochondrial complexes (I and II), leading to apoptosis and necrosis [26]. In the other model, Glu induces potentially lethal oxidative damage by disruption of the redox system. Both models in the SH-SY5Y cell line have been previously used in neuroprotection studies [26,27].

Cytoprotective and/or antioxidant activities related to degenerative disorders of **K**, **iP**, **BAP**, **iPR**, ***t*ZR,** and their free bases have been tested, and (as outlined above) some CKs have been found to have protective activities in neuronal cells. However, no previously published studies have examined the structure-neuroprotective activity relationship (SAR) of natural CKs (Table 1). Therefore, this study was undertaken to examine neuroprotective (anti-parkinsonian) activities of almost all known naturally occurring CKs in the selected SAL- and Glu-induced models of neurodegeneration. First, we evaluated each of the CKs’ oxygen radical absorbance capacity (ORAC) and (in safety tests) cytotoxicity towards neuron-like SH-SY5Y cells. Then, we evaluated the compounds’ neuroprotective effects and influence on oxidative stress levels by measuring superoxide (O_2_^.^) production (dihydroethidium, DHE assay) and apoptotic caspase-3,7 activities. The results provide the first reported systematic indications of the relationships between natural CKs’ structures and neuroprotective activities.

## 2. Results and Discussion

### 2.1. Cytokinins’ Oxygen Radical Absorbance Capacity (ORAC)

As neurodegenerative diseases are associated with elevated oxidative stress, antioxidant activity plays a key role in defenses of neuronal cells. To assess CKs’ biological potential in this respect, antioxidant capacity was determined by ORAC, which is commonly used to determine substances’ antioxidant capacity [28]. Antioxidant capacity was expressed as Trolox equivalents (TE), which determines the effectiveness (lower to higher) of compounds than Trolox on equimolar basis. The results, presented in Table 1, show that topolins (***o*T**, ***m*T** and ***p*T**) and their ribosides (***o*TR**, ***m*TR**, ***p*TR**) have high antioxidant activities, which are probably closely related to the electron-rich system of their C6-hydroxybenzylamino substituent. Despite their high ORAC values, the topolins did not have high neuroprotective activity. However, several heteroaromatic CKs including **K** (N^6^-furfurylaminopurine) and non-aromatic *cis*-zeatin-O-glucoside (***c*ZOG**), which has a 4-hydroxy-3-methylbut-2-en-1-yl)amino substituent, also showed high antioxidant capacity (Table 2). Other CK metabolites—including kinetin-3-glucoside (**K3G**), kinetin riboside 5′-monophosphate (**KMP**), kinetin-9-glucoside (**K9G**), and *trans*-zeatin riboside-5′-monophosphate (***t*ZMP**)—had moderate antioxidant activity. All the others had detectable capacity except **BAP**. These results confirm previous findings that **iP**, ***p*T**, **K** can act as direct radical scavengers, but conflict with previously reported activity of BAP in the ORAC test [20,21]. To conclude, these compounds have potential in the treatment of neurodegenerative diseases associated with increased oxidative stress [29].

### 2.2. Differentiation of SH-SY5Y Cells

**Figure 1:**
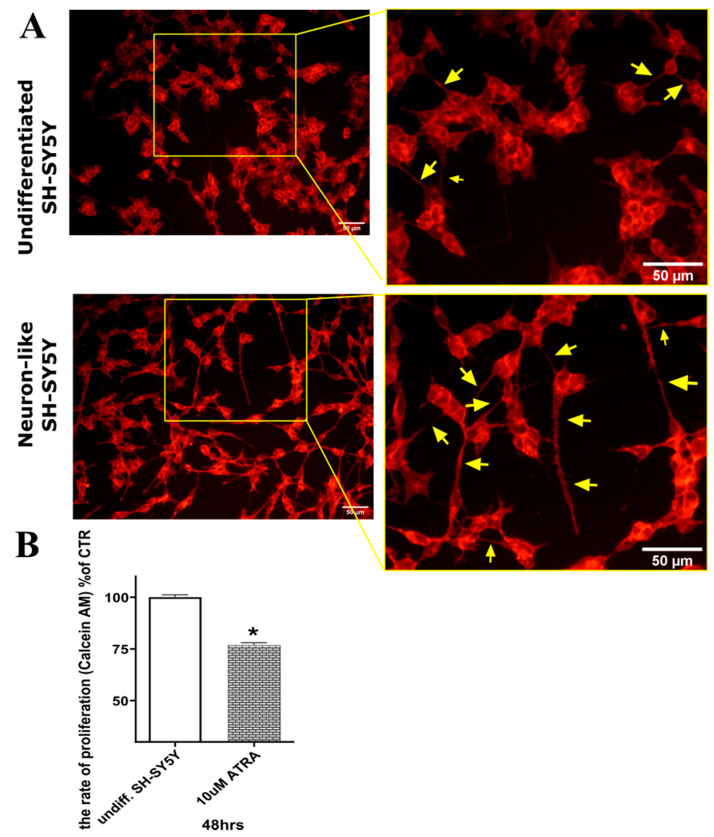
(**A**) Fluorescent micrographs of SH-SY5Y cells with membranes stained using a Neurite outgrowth kit (Invitrogen™): Control, undifferentiated cells (exposed to mock treatment solution: <0.1% DMSO); Cells differentiated by exposure to 10 µM all-trans retinoic acid (ATRA) for 48 h. Bars = 50 μm. (**B**) Proliferation rates of undifferentiated and differentiated SH-SY5Y cells: numbers of viable cells after 48 h exposure to <0.1% DMSO and 10 µM ATRA, respectively. Data were obtained from five independent experiments with triplicate cultures: asterisks show the significance of differences in numbers of viable cells (as percentages of numbers of undifferentiated cells) between the cultures: * *p* < 0.05.

To study CKs’ neuroprotective effects, SH-SY5Y neuroblastoma cells (chosen for reasons already described [23]) were differentiated by exposure to 10 µM ATRA for 48 h as previously described [23,24]. They were then stained using a membrane staining kit to examine morphological differences between undifferentiated and differentiated cells. As shown in Figure 1A, the neuron-like differentiated cells grew less densely, were more prolonged, and produced more neurites (indicated by yellow arrows in the figure) than the undifferentiated cells. These morphological changes associated with differentiation have been previously observed, even after shorter exposure (24 h) to ATRA [24,30]. More importantly, the number of neurites rises dramatically to a level when they can create a neurite network. From this reason, cell viability was measured to compare the rate of proliferation of undifferentiated and differentiated SH-SY5Y cells. The viability of undifferentiated SH-SY5Y was taken as the maximum rate of proliferation. The results present in Figure 1B show that proliferation rate (assessed by Calcein AM viability assay) of SH-SY5Y was reduced by 23% after 48 h ATRA treatment.

### 2.3. Cytotoxicity of Cytokinins towards Neuron-like SH-SY5Y Cells

In tests of the CKs’ potential cytotoxicity with the Calcein AM viability assay [31] most showed low toxicity towards the neuron-like SH-SY5Y cells. The decrease of viability below 90% was considered as threshold for neurotoxic effect. The only two exceptions were **KR** (11.9%) and ***p*TR** (10.5%), in accordance with previous findings that some cytokinin metabolites, particularly ribosides, may have cytotoxic effects [32]. Other ribosides, such as ***c*ZR**, **iPR**, ***o*TR**, ***m*TR**, caused no apparent reduction in the neuron-like SH-SY5Y cells’ viability (Table 3). **DFO** [33,34] and **NEC-1** [35,36] used as positive controls in our in vitro model were also proved by other studies on SH-SY5Y cells to be non-toxic. In conclusion, mainly derivatives **KR** and ***p*TR** showed lower viability than 90% and were therefore considered less interesting for further evaluation in both in vitro models of neurodegeneration.

### 2.4. Identification of Neuroprotective Cytokinins in the SAL-induced Model of PD

**Figure 2:**
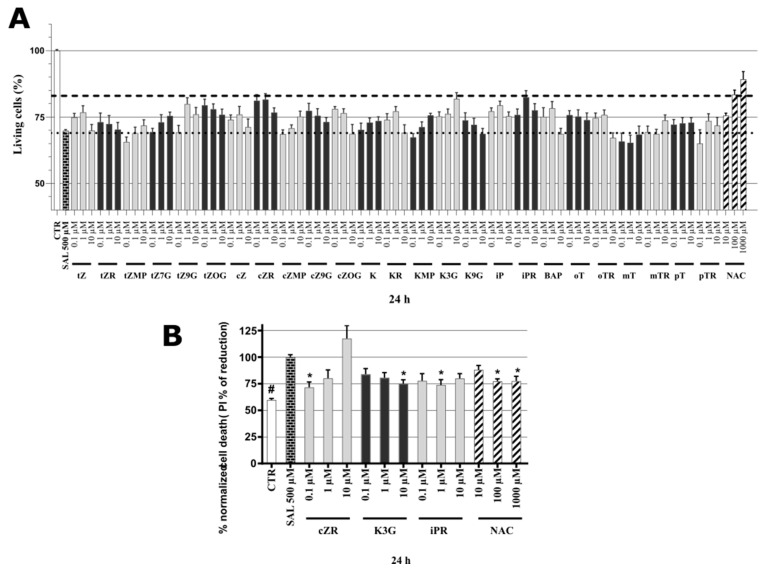
(**A**) Neuroprotective activity of cytokinins and *N*-acetylcystein (**NAC**) in SAL-induced model of PD on neuron-like SH-SY5Y cells. The dashed line shows the **NAC** effect threshold at which cytokinins were selected for further testing; the dotted line then counts the number of living cells in the Calcein AM assay after treating the cells with 500 µM SAL; healthy control cells (CTR, DMSO < 0.1%). Triplicates in at least three separated days. (**B**) Normalized SH-SY5Y cell death after propidium iodide staining. Triplicates in at least five independent days. * *P* compared with vehicle with 500 µM SAL, # *P* compared with vehicle without 500 µM SAL.

For these tests, neuronal SH-SY5Y cells were differentiated for 48 h then co-treated with 500 µM SAL and each CK at three concentrations (0.1, 1, 10 µM). As shown by the dotted line in Figure 2A, application of the neurotoxin SAL at 500 µM reduced the viability of differentiated SH-SY5Y cells, according to the Calcein AM assay, by 30%. *N*-acetylcysteine (**NAC**) was used as a positive control in these tests due to its previously reported neuroprotective effect in the same SH-SY5Y cell-based in vitro model [37]. Concentrations of 10, 100, and 1000 μM **NAC** were used to induce partial or almost complete recovery in the SAL-model. **NAC** was able to increase cell viability at 100 μM and 1 mM concentration, corresponding to 83.39 ± 1.74% and 89.21 ± 2.89%, respectively. **NAC**’s protective activity at 100 μM (indicated by the dashed line in Figure 2A) was used as a potency threshold for selecting CKs for further tests. According to this setup, the biologically significant neuroprotective activities have been observed with **K3G** at 10 µM (81.84 ± 2.36%), ***cZR*** at 0.1 µM (81.14 ± 2.30%) and 1 µM (81.53 ± 2.24%) and **iPR** at 1 µM (82.43 ± 2.51%). Thus, **iPR** and **cZR** were effective neuroprotectants at lower micro or sub-micromolar concentrations than **NAC**. The cytokinin screening also revealed that many other metabolites can moderately increase the viability of differentiated SH-SY5Y cells exposed to SAL. However, some tested CKs (including ***t*ZR**, ***t*ZMP**, ***m*T**, ***m*TR**, **pT,** and ***p*TR**) had very little protective effect.

To confirm the most active natural CKs’ anti-PD activities, overall cell death rates were quantified by propidium iodide (PI) staining, which (in contrast to cell metabolism-based viability tests) only labels cells with impaired membrane integrity, dying cells, and already dead cells [38]. Results were normalized with respect to the cell death rate following treatment with SAL alone (set as 100%). As shown in Figure 2B, the **NAC** positive control substance significantly reduced cell death rates at both 100 and 1000 µM (to 77.3 ± 2.21% and 77.5 ± 4.44%, respectively). Overall, **NAC** proved to be a neuroprotective agent with comparable activities to those recorded in other studies in a dose-dependent manner (in the 50–500 µM range) for SH-SY5Y cells [37]. The PI assay also showed that the CKs ***c*ZR**, **K3G**, and **iPR** have protective activities, especially ***c*ZR,** which reduced the cell death rate to 71.6 ± 5.08% at 0.1 μM. In contrast to ***c*ZR**, **K3G** had reversed dose-dependent effects, with maximum activity at 10 μM (reducing the cell death rate to 75.0 ± 3.69%) and **iPR**’s activity peaked at 1 μM (reducing the rate to 73.9 ± 4.99%). Taken together, as shown in Figure 2, CKs provided comparable neuroprotective activity to 100 µM **NAC** according to both the viability and cytotoxicity assays. Moreover, effective concentrations of CKs such as ***c*ZR** and **iPR** were much lower than those of **NAC**, in the sub-micromolar and micromolar ranges. Previous observations obtained following double staining with PI and annexin V/PI indicate that **K** may reduce apoptosis [39], thus we also investigated effects of CKs and **NAC** on oxidative stress and caspase-3,7 activation (a well-known apoptosis marker).

### 2.5. Cytokinins Decrease SAL-induced Superoxide Radical Formation

**Figure 3:**
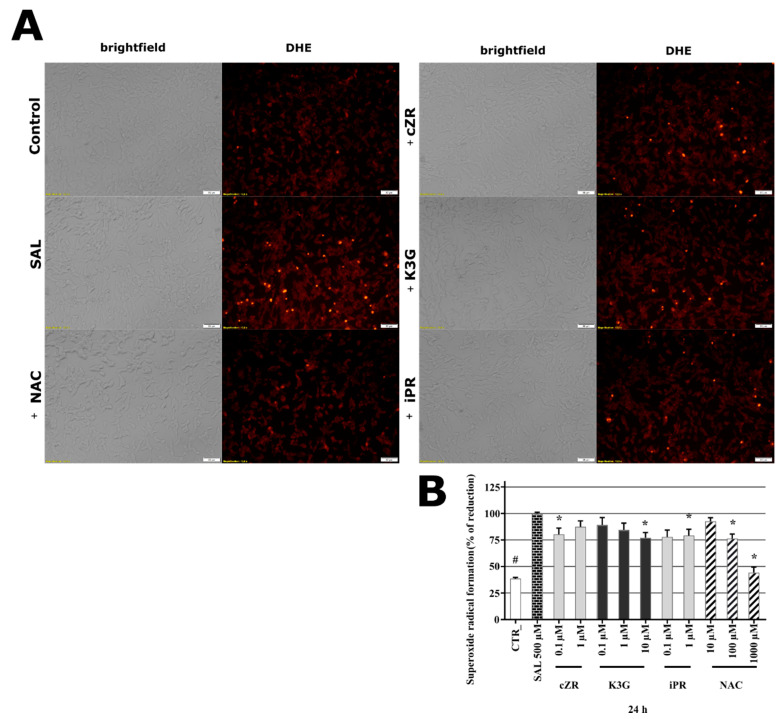
(**A**) Microphotographs showing SAL-induced oxidative stress and oxidative stress-reducing activities of cytokinins in human differentiated neuron-like SH-SY5Y cells visualized by fluorescence microscopy following dihydroethidium (DHE) labelling. Bars = 50 μm. The images show cells treated with DMSO solution (controls), 500 µM salsolinol (SAL) alone, and combinations of 500 µM SAL and 1000 µM **NAC** (+**NAC**), 0.1 µM ***c*ZR** (+***c*ZR**); 10 µM **K3G** (+**K3G**), 1µM **iPR** (+**iPR**) for 24 h before staining with DHE. (**B**) SAL-induced superoxide radical formation and cytokinin or *N*-acetylcysteine (**NAC**) protective activity. The graph shows the quantification of DHE stained cells using Infinite M200 Pro microplate reader (Tecan, Austria). Triplicates in at least five independent days. * *P* compared with vehicle with 500 µM SAL, # *P* compared with vehicle without 500 µM SAL.

Oxidative stress (OS) is a key pathological contributor to several neurodegenerative diseases, and both SAL (at > 100 µM) and tetrahydroisoquinolines are potent OS inducers [26,40]. Thus, we also measured formation of superoxide (a ROS and important OS marker) in the presence of SAL with and without selected CKs or **NAC**. To ensure that SAL caused sufficient OS damage in SH-SY5Y cells to detect responses, cells were exposed to 500 µM SAL for 24 h, as in previous work [37] and in accordance with findings presented above. The cells were then stained by dihydroethidium (DHE) to detect superoxide radical formation [41,42]. As can be seen in Figure 3A, cells were visually observed after labelling with DHE (which provides red fluorescence signals following reaction with superoxide). SAL induced a clear rise in DHE fluorescence, relative to levels in control and **NAC**-treated cells. Moreover, three CKs (***c*ZR**, **K3G,** and **iPR**) had similar visual effects to **NAC** (100 µM) on DHE fluorescence. Furthermore, the spectrophotometric quantification with respect to levels detected in cells treated by SAL alone (set as 100%), was in line with microscopy observation. As shown in Figure 3B, the normalized superoxide level in healthy control cells (CTR) was less than 39%, and the positive control substance **NAC** provided moderate-to-complete reduction of SAL-induced ROS production at 100 and 1000 µM (to 76.3 ± 4.33 and 44.3 ± 5.12%), suggesting that glutathione (GSH) depletion plays a key role in the model [26]. Interestingly, SAL induced dramatic reductions in GSH contents of SH-SY5Y cells accompanied by elevation of OS, to levels similar to those previously observed in a study that also recorded **NAC**-mediated effects on cell viability, cell death, and glutathione contents [43]. Results presented here show that **NAC** also reduced superoxide radical formation to basal levels (i.e., levels in DMSO-treated controls). CK ribosides were tested at active concentrations (0.1–1 µM) along with **K3G**, and significantly reduced the cells’ superoxide radical contents to the following levels (relative to those of cells treated with SAL alone): ***c*ZR** 80.34 ± 5.99% at 0.1 µM; **K3G** 77.1 ± 4.89% at 10 µM; **iPR** 79.2 ± 5.91% at 1 µM, comparable to the effects of 100 µM **NAC**. Collectively, the orthogonal demonstrations strongly indicate that potent anti-OS activity plays a key role in the protective effects of **NAC** and CKs in the SAL-induced PD model. A correlation between OS amelioration and neuroprotection has also been noted by other authors [29], and several studies have found that **K** and **BAP** can directly ameliorate OS activities [44] through formation of complexes with Cu^2+^ ions, resulting in superoxide dismutase-like activity [45,46]. However, CKs have also been described as indirect antioxidants with effects mediated by induction of the nuclear factor erythroid 2-related factor 2 (NRF2) antioxidant response pathway (**iPR**) [22] or partial restoration of glutathione peroxidase and SOD activities (**K**) [16]. In addition, **K** reportedly has neuroprotective activities against OS injury induced by H_2_O_2_ in SH-SY5Y cells [17]. Both types of reported anti-ROS activity of CKs could potentially explain to effects of ***c*ZR**, **K3G,** and **iPR** in reduction of superoxide radicals in the SAL-induced SH-SY5Y cell PD model [47,48,49,50].

### 2.6. Anti-Apoptotic Effects of Cytokinins Determined by Caspase-3/7 Activity Measurements

**Figure 4:**
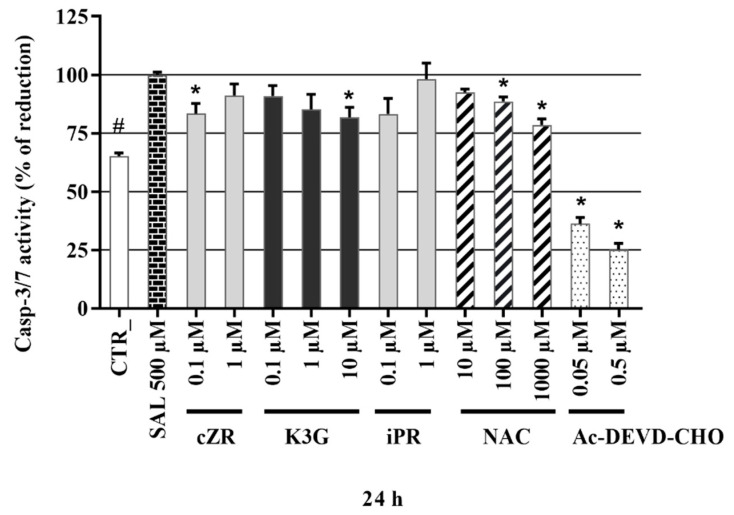
Caspase-3/7 activity in the SAL-induced model of PD. Triplicates in at least four independent days. * *P* compared with vehicle with 500 µM SAL, # *P* compared with vehicle without 500 µM SAL.

As shown by the PI staining assays described above, SAL induced increases in the SH-SY5Y cells’ death rates. As SAL is associated with both apoptosis and necrosis [51] we also investigated the activation of caspase-3 and 7 (casp-3/7) as a specific marker of apoptosis (the execution phase) [52] after exposing the cells to CKs. Caspase-3/7 activities recorded following treatment with each of the test compounds were normalized with respect to those recorded following treatment with 500 µM SAL (set as 100%). As shown in Figure 4, the well-known caspase inhibitor **Ac-DEVD-CHO** (included as a specific, apoptosis-related control) strongly inhibited caspase-3/7 activity at sub-micromolar concentrations (to 36.3 ± 2.66 and 25.2 ± 2.69% of levels in cells treated with SAL alone at 0.05 and 0.5 μM, respectively). Similar levels of inhibition have been previously observed [53] in different in vitro SH-SY5Y cell-based models of neurodegeneration. The positive control **NAC** also significantly reduced caspase-3/7 activity, to 88.7 ± 1.87% and 78.5 ± 2.56% of SAL-induced levels at 100 μM and 1000 μM, respectively. The tested CKs had a wide array of effects on caspase-3/7 activities. Interestingly from the CK ribosides, only ***c*ZR** had significant effects on the activities at 0.1 µM (reducing them to 83.3 ± 4.33% of SAL-induced levels, respectively), while **iPR** at 1 µM did not significant reduction of casp-3,7. Finally, **K3G** decreased caspase-3/7 activity with maximum effect at 10 μM (to 81.7 ± 4.31% of the SAL-induced level). Overall, SAL-induced a 1.6-fold increase in caspase-3/7 activity compared to healthy control cells (CTR, Figure 4), in accordance with results of an earlier study, in which a similar SAL concentration (400 µM) and the same cell line were used [54]. **NAC** is known to influence caspase-3/7 activity in several models of neuronal death [55,56,57,58]. However, results presented here provide the first demonstration of their caspase-3/7 in an SH-SY5Y cell-based SAL-induced model of neuronal death. The positive control reduced the caspase-3/7 activity induced by 500 µM SAL in a similar manner to CKs, but ***c*ZR** and **K3G** were effective even at sub-micromolar or micromolar concentrations. Other studies with stress models associated with PD (proteasome inhibitor MG 132- or H_2_O_2_-induced toxicity in SH-SY5Y cells [17] and fibroblasts [15]) and Huntington’s disease (serum starvation model in PC12 cells [19]) have also shown that the CKs **K** and ***t*ZR** can have anti-apoptotic (caspase-3) activities [17], and that both **K** and ***t*ZR** can have anti-senescence activities [17,19]. Moreover, associations between decreasing of casp-3,7 activation and neuroprotective activity was reported for SAL and SAL-related models [54,59,60].

### 2.7. Identification of Cytokinin Neuroprotective Activity in the Glutamate-induced Cell Death Model

**Figure 5:**
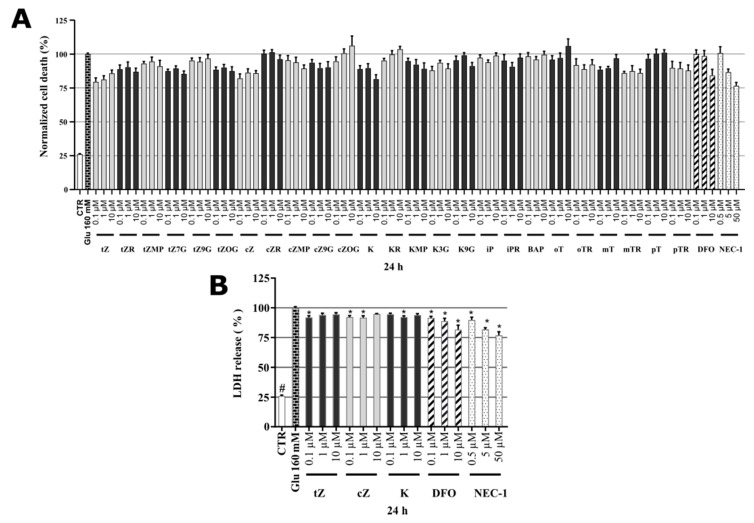
(**A**) Death rate of neuron-like SH-SY5Y cells in glutamate (Glu)-induced model of cell death; (**B**) Glu-induced toxicity (LDH-release) of neuron-like SH-SY5Y cells. Triplicates in at least three independent days.* *P* compared with vehicle with 160 µM Glu, # *P* compared with vehicle without 160 µM Glu.

As SH-SY5Y cells do not express all NMDA receptor subunits [61], the most likely mechanism of glutamate (Glu) toxicity is blockage of the cystine/glutamate Xc-antiporter with massive induction of oxidative stress [62]. Previous authors have already demonstrated an association between glutamate-induced cell death and necroptosis in SH-SY5Y cells [63], and the importance of caspase-3 activation in glutamate-induced toxicity towards them [62,64]. Several studies have also shown the occurrence of iron-driven cell death (ferroptosis) after glutamate intoxication [62,65,66], and indicated the occurrence of three types of cell death (necroptosis, apoptosis, and ferroptosis) following blockade of the Xc-antiporter system. In order to complete a comprehensive evaluation of the neuroprotective potential of the tested cytokinins, the glutamate-induced cell death assay system was included in an analytical panel with the known neuroprotective iron chelator deferoxamine (**DFO**) and inhibitor of necroptosis necrostatin-1 (**NEC-1**) [67] as positive controls. Here, SH-SY5Y cells were differentiated for 48 h, then treated with 160 mM Glu or in co-treatment thereof with various cytokinin concentrations (0.1–10 μM) for 24 h and stained by PI. In this model, the effect of Glu on cell death was 100% PI signal, so the reduction in cell death by the test compounds was evaluated. Glu induced almost a 4-fold increase in cell death compared to healthy SH-SY5Y controls (26.1 ± 0.42%). Screening of positive controls and cytokinins revealed that both positive controls **NEC-1** (50 μM, 76.6 ± 2.51%) and **DFO** (10 μM, 84.2 ± 4.54%) decreased the cell death induced by glutamate. As shown in Figure 5A, from the panel of cytokinins, only three cytokinins were able to protect neuron-like SH-SY5Y cells in similar fashion as positive controls. The most active cytokinins were *trans*-zeatin (***t*Z)** (0.1 μM, 79.5 ± 2.91%; 1 μM, 81.3 ± 2.77%) and cis-zeatin (***c*Z**) (0.1 μM, 82.1 ± 3.29%; 1 μM, 86.2 ± 2.89%) and kinetin (**K**) (1 μM, 88.0 ± 3.76%; 10 μM, 79.9 ± 3.44%). To confirm their promising activity, an orthogonal assay was used to elucidate in more detail CKs’ biological effects in this model: A lactate dehydrogenase (LDH)-release assay [68], which also showed a dramatic (4-fold) increase in toxicity following Glu treatment. Interestingly, the determination of LDH-release allowed us to differentiate between activities of CKs and the positive controls (**NEC-1** and **DFO**). The CKs had moderate but significant protective activity (***t*Z** reduced the death rates to 91.9 ± 1.40% at 0.1 µM; ***c*Z** reduced them to 92.3 ± 1.15% at 0.1 µM and 91.7% ± 1.56% at 1 µM; **K** reduced them to 92.2 ± 1.06% at 1 µM). The positive controls **NEC-1** and **DFO** had stronger protective effects but were effective at much higher concentrations (reducing the rates to 76.9 ± 2.94% and 81.6 ± 3.74% at 50 and 10 μM, respectively) (Figure 5B). The LDH assay validated the indications obtained with PI staining and observed effects of the positive controls were consistent with published data [69,70]. Findings regarding effects of **K** in Glu-induced cell death model in HT22 cells [18] and our observations of effects of representative CKs such as ***t*Z**, ***c*Z,** and **K** show that CKs are promising candidates for treating neurodegenerative diseases associated with oxidative stress [71]. Finally, ***t*Z** and ***c*Z** were preferred because they were effective at lower concentrations and were selected, together with **K**, for further studies of their effects on levels of oxidative stress and caspase-3,7 activation.

### 2.8. Effects of Cytokinins on Glu-induced Oxidative Stress in SH-SY5Y cells

**Figure 6:**
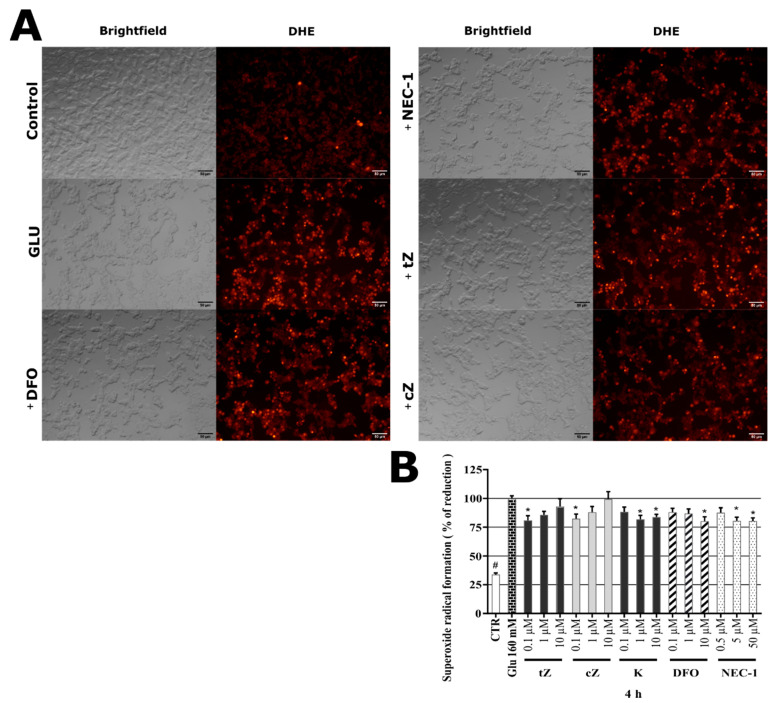
(**A**) Glu-induced oxidative stress (OS) and OS-reducing activity of indicated compounds in human neuron-like SH-SY5Y cells visualized by fluorescence microscopy following dihydroethidium (DHE) labelling. Bars = 50 μm. Images show neuron-like SH-SY5Y cells treated by DMSO (Control), 160 mM glutamate (Glu) alone, and together with: 10 µM deferoxaine (+**DFO**), 50 µM necrostatin-1 (+**NEC-1**), 0.1 µM ***t*Z** (+***t*Z**) and 0.1µM ***c*Z** (+***c*Z**) for 4 h, then stained by DHE. (**B**) Glu-induced superoxide radical formation in neuron-like SH-SY5Y cells after 4 h. The graph displays the quantification of DHE stained cells using Infinite M200 Pro microplate reader (Tecan, Austria). Triplicates in five independent days. * *P* compared with vehicle with 160 µM Glu, # *P* compared with vehicle without 160 µM Glu.

Unlike the previous model, Glu can induce oxidative stress in SH-SY5Y cells by different pathways. One is based on blockage of the Cystine/glutamate (Xc-) antiporter, which leads to glutathione (GSH) depletion and negative effects on superoxide dismutase (SOD) activity [62]. Glu-mediated cell death also putatively involves Rac-NADPH-oxidase-driven ROS (particularly superoxide radical) formation in SH-SY5Y cells [72]. These findings indicate that the primary cause of cell death in the Glu-induced model is OS. Within the model, neuron-like cells were co-treated with Glu and tested compounds, stained by DHE, and observed by fluorescence microscopy. As can be seen in Figure 6A, a dramatic rise in red DHE fluorescence was observed in Glu-treated cells, which was ameliorated by the positive control substances and tested CKs. Based on the microscopy observation, the quantification of superoxide levels was performed in Glu-induced cells in a similar manner, by the DHE assay, to those in SAL-induced cells. The results were normalized with respect to levels induced by Glu alone (set as 100%), which caused a dramatic, almost 3-fold increase in superoxide levels within just 4 h in neuron-like SH-SY5Y cells relative to levels in healthy control cells (CTR: 33.79 ± 1.45%). As shown in Figure 6B, CKs such as ***t*Z**, ***c*Z,** and **K** significantly reduced the superoxide level in Glu-intoxicated cells: 0.1 μM ***t*Z**, 0.1 μM ***c*Z**, 1 μM and 10 μM **K** reduced it to 81.0 ± 4.03, 80.6 ± 3.89, 81.8 ± 3.39 and 83.8 ± 2.32% of levels in cells treated with Glu alone. These levels are comparable to those obtained with the necroptosis inhibitor **NEC-1** (80.5 ± 3.19% at 5 μM and 80.4 ± 2.70% at 50 μM) and iron-chelator **DFO** (80.2 ± 3.80% at 10 μM). The results obtained with the Glu-induced cell death model, particularly the effects of positive controls, are consistent with previous reports [70,73]. Previous evidence that CKs have OS-reducing effects in Glu-induced or similar models is limited to a demonstration that **K** is a potent OS-reducing agent (at approximately 23 µM) in the HT22 cell-based Glu-induced cell death model [18]. We found that **K** had an OS-reducing effect at lower concentrations (1–10 µM) in our SH-SY5Y cells. In addition, beneficial long-term effects of ***t*Z** on human fibroblasts, including hydrogen peroxide-decomposing activity, have been observed [15], suggesting that ***t*Z** and ***c*Z** may have indirect OS-reducing effects in Glu-induced cell death models. Additionally, other reports pointed out the associations between CKs-mediated OS-reducing effect and protective activity [22,74,75,76]. Taken together, ***t*Z**, ***c*Z,** and **K** showed surprisingly strong OS-reducing effects, comparable to those of positive controls, despite differing strengths in overall neuroprotective effect.

### 2.9. Effects of Cytokinins on Caspase-3/7 Activity in the Glu-induced Cell Death Model

**Figure 7:**
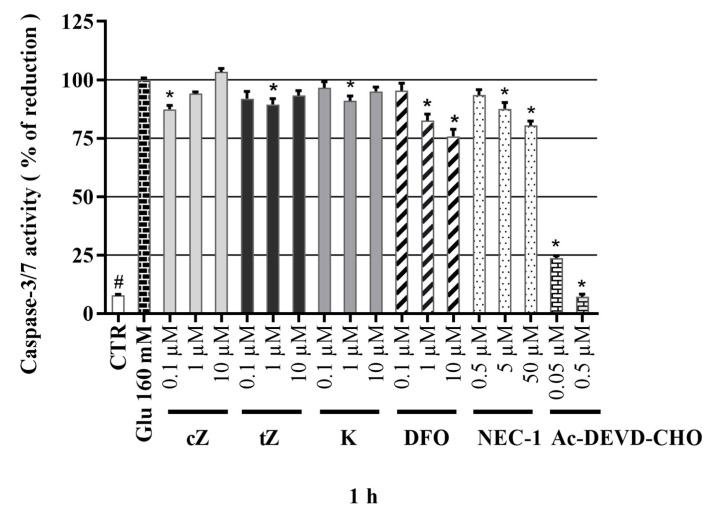
Caspase-3/7 activity in Glu-model of oxidative damage of neuron-like SH-SY5Y cells. Triplicates in at least four independent days. * *P* compared with vehicle with 160 µM Glu, # *P* compared with vehicle without 160 µM Glu.

Glu has similar toxic effects on SH-SY5Y cells to previously reported effects on HT22 cells associated with Xc-antiporter blockage. Thus, non-apoptotic cell death mechanisms (ferroptosis, necroptosis, etc.) are involved in both cases [62] although expression of caspase-3 and to some degree the NMDA subunit of NR1 may be more strongly occurred in SH-SY5Y cells [61]. Hence, final stages of cell death in the two cell lines may differ. A contributory role of caspase-3 activity has been observed in many studies in the Glu-induced model of cell death of SH-SY5Y cells [62,77,78,79]. In this assay, neuron-like SH-SY5Y cells were treated here with 160 mM Glu, which leads to elevation of caspase-3/7 activity. In efforts to elucidate the role of apoptosis in Glu-induced death of SH-SY5Y cells, a specific caspase-3/7 inhibitor, **Ac-DEVD-CHO**, was applied and found to have strong inhibitory effect: At 50 nM it reduced caspase-3,7 activity to 23.70 ± 1.01% of the level in cells treated with Glu alone and at 0.5 µM it almost completely blocked the activity (reducing it to a normalized level of just 7.22 ± 1.05%; comparable to the level observed in healthy cells: 7.8 ± 0.39%), as shown in Figure 7. Application of positive controls resulted in a gradual dose-dependent decrease in caspase-3,7 activity. Interestingly, **NEC-1** had a slight effect with maximum effect at 50 μM and **DFO** had more robust inhibitory activity at 10 μM (reducing the activity to 80.5 ± 1.85 and 75.8 ± 3.00%, respectively). On the other hand, only slight, but significant effects on casp-3,7 were achieved by ***tZ*** at 1 µM (89.5 ± 2.50%), ***cZ*** at 0.1 µM (87.4 ± 1.62%) and **K** at 1 µM (91.0 ± 2.06%). Taken together, the results show that both positive controls had stronger caspase-3/7 activity-decreasing activity than the tested CKs, but the CKs had effects at much lower concentrations. Overall, the results indicate that modulation of caspase-3/7 activity plays a key role in the potent neuroprotective activity of agents such as **DFO** [58] in the Glu-induced model of cell death [79,80]. However, as shown in this section, **NEC-1** also had neuroprotective activity, suggesting that other types of cell death might be involved in Glu-induced death of neuron-like SH-SY5Y cells [63,67,81]. Indications that caspase-independent mechanisms are involved in Glu-induced cell death have also been obtained in analyses of effects of **NEC-1** [70], **DFO** [69], and K [18] on HT22 cells.

## 3. Conclusions

In summary, our study revealed that CKs and their metabolites have neuroprotective activities in the SAL-induced model of Parkinson’s disease and Glu-induced oxidative damage in human differentiated SH-SY5Y neuronal cells. **K3G**, ***c*ZR,** and **iPR** were found to have biologically significant neuroprotective activities. Moreover, the active CKs were effective at lower (sub-micromolar and micromolar) concentrations than the positive control substance **NAC**. **K3G, *c*ZR** and **iPR** also had positive effects on viability, cytotoxicity, oxidative stress and caspase-3,7 activity (except **iPR**). The orthogonal demonstrations strongly indicate that anti-oxidative stress activity plays a key role in CKs’ protective effects in the SAL-induced cell death model. Only three metabolites in the tested panel of CKs (***tZ***, ***c*Z,** and **K**) protected neuron-like SH-SY5Y cells in the Glu-induced model of oxidative damage in a similar fashion to the iron chelator **NEC1** and necroptosis inhibitor **DFO**. To confirm their promising activity, we tested their effects in an orthogonal lactate dehydrogenase (LDH)-release assay. The CKs had moderate but significant protective activity. This was weaker than corresponding activities of the control substances, but despite the differences in modulation of neuronal health, all three tested CKs stimulated potent reductions in superoxide radical formation, similarly to the positive control substances. The CKs also had comparable effects on Glu-induced oxidative stress in SH-SY5Y cells to those of **NEC1** and **DFO**, but these compounds had stronger inhibitory effects on caspase-3/7 activity than the CKs. As Glu-induced cell death does not rely on a caspase-dependent pathway, as shown in studies of effects of **DFO** and **NEC-1** on HT-22 cells [70], CKs can apparently modulate both caspase-dependent and -independent cell death by reducing oxidative stress [18,82]. In ongoing studies, the detailed mechanism of action of CKs and/or their mitochondrial effects on neuronal or astrocyte cells will be investigated.

## 4. Materials and Methods

### 4.1. Chemicals and Reagents

Cytokinin standards were obtained from OlChemIm (Olomouc, Czech Republic). Calcein AM (1 mg/mL solution), an LDH release kit and a Neurite outgrowth kit were purchased from ThermoFisher. Propidium iodide, dihydroethidium, components of caspase-3/7 assay buffer, DMEM/F12 1:1 medium, fetal bovine serum, trypsin, ATRA, salsolinol hydrobromide, glutamate monosodium salt, deferoxamine, *N*-acetylcysteine, Ac-DEVD-CHO, and DMSO for cell cultures were purchased from Sigma Aldrich (Merck). Ac-DEVD-AMC substrate was supplied by Enzo Life Science.

### 4.2. ORAC Radical Scavenging Activity Assays

Compounds’ ability to scavenge free radicals in vitro was determined by the Oxygen Radical Absorbance Capacity (ORAC) assay. Briefly, fluorescein (100 µL, 500 mM) and 25 µL of tested compound solution was added to a 96-well microplate pre-incubated at 37 °C. Next, 25 µL of 250 mM 2,2′-azobis(2-amidino-propan)dihydrochloride (AAPH) was quickly added, the microplate was shaken for 5 s and red fluorescence (with 485 and 510 nm excitation and emission wavelengths, respectively) was measured every 3 min over 90 min using an Infinite 200 microplate reader (TECAN, Männedorf, Switzerland). NAUC (Net Area Under Curve) values were used to express antioxidant activities relative to that of Trolox (a synthetic hydrophilic analogue of α-tocopherol, vitamin E). Substances with ORAC values greater than zero are deemed to actively trap free radicals.

### 4.3. SH-SY5Y Cell Culture

The human neuroblastoma cell line SH-SY5Y purchased from ATCC (American Type Culture Collection, Manassas, VA, USA) was grown in Dulbecco’s modified Eagle’s Medium and Ham’s F12 Nutrient Mixture (DMEM:F-12, 1:1), supplemented with 10% fetal bovine serum (FBS) and 1% penicillin and streptomycin at 37 °C in a humidified 5% CO_2_ atmosphere. Cells were passaged up to 20 times and media were changed twice or thrice a week. Cell density was set as appropriate for the planned assay (5000, 7000, 10,000, or 20,000 cells per well) in 96-multiwell plates in 100 μL total volumes of medium for each experiment. The day after the seeding, ATRA in 1% FBS DMEM/F12 medium was added to the cells to a final concentration of 10 µM and incubation was continued for a further 48 h to induce differentiation, allow formation of longer neurites, and reduce proliferation.

### 4.4. Microscopy

Micrographs of neuron-like SH-SY5Y cells were obtained using a DM IL LED fluorescence microscope (Leica Microsystems, Mannheim, Germany) with an appropriate excitation filter for the assay, or a brightfield setup with a DP73 high-performance digital camera (Olympus, Tokyo, Japan). Since the signal-to-noise ratio for staining was moderate, the contrast was slightly adjusted in ImageJ software (Fiji) without affecting the resulting observation. Original images are included in the Supplementary Materials.

### 4.5. Cell Membrane Staining (Neurite Outgrowth kit, Invitrogen™)

Neuron-like SH-SY5Y cells (5000 cells per well) obtained by the 48 h differentiation procedure were stained by a Neurite outgrowth kit (Invitrogen™) according to the manufacturer’s recommendations with minor modification. The cells were washed with PBS, labelled with a solution of the membrane staining dye supplied with the kit (following the protocol for 96 multiwell plates) in PBS for 20 min at 37 °C, washed again with PBS and viewed under a fluorescence microscope (with 533 and 585 nm excitation and emission wavelengths, respectively).

### 4.6. Cell Treatment

After the differentiation procedure, the differentiation media was changed to 1% DMEM/F12 medium supplemented with test compounds at concentrations of 0.1, 1, and 10 μM (with 7000 cells per well for cytotoxicity tests), or together with the toxin SAL at 500 µM (with 7000–10,000 cells per well) or 160 mM Glu (with 20,000 cells per well) for an appropriate duration for the assay type (viability, cell death, etc.). Control cells were treated with medium containing ≤ 0.1% of DMSO.

### 4.7. Cell Viability and Cell Death

The viability of neuron-like SH-SY5Y cells growing in 96-well microplates (with ca. 7000 cells per well) after 24 h of treatments was evaluated by the Calcein AM assay with minor modification [31]. The Calcein AM solution in PBS used had 0.75 µM concentration and the incubation time was set to 50 min. The number of living cells in each well was determined using an Infinite M200 Pro microplate reader (Tecan, Austria), with 495 and 517 nm excitation and emission wavelengths, respectively.

Cell death of neuron-like cells (SAL model: 10,000 cells per well; Glu-model: 20,000 cells per well) was determined by PI assay as reported by Stone et al. 2003 with small modification [83]. Briefly, the medium of SAL-induced model was aspirated and changed to a 1 µg/mL solution of PI in PBS, but cells subjected to Glu-induction have weaker adherence so a 1 mg/mL solution of PI in PBS was added to give a final concentration of 1 µg/mL. In both cases, cells were incubated for a further 15–25 min at room temperature then PI fluorescence was measured by an Infinite M200 Pro reader (Tecan, Grödig, Austria) with 535 and 617 excitation and emission wavelengths, respectively. The resulting PI fluorescence obtained with toxins alone was set as 100% cell death.

### 4.8. Measurement of Oxidative Stress by the Dihydroethidium (DHE) assay

Cells were differentiated and treated as described in the previous section for SAL-induction (10,000 cells per well, 24 h or Glu-induction (20,000 cells per well, 4 h). After the treatments, cells were centrifuged at 500× *g* for 330 s, then the culture media were replaced (following aspiration) with 10 μM DHE solution in PBS. 96-multiwell plates with cells were incubated in the dark for 30 min at room temperature, then DHE fluorescence was measured by an Infinite M200 Pro microplate reader (Tecan, Grödig, Austria) with 500 and 580 nm excitation and emission wavelengths, respectively. The resulting DHE fluorescence obtained with SAL or Glu alone was set as 100% superoxide radical formation. Illustrative fluorescence microphotographs of DHE-stained cells were obtained after DHE measurement with the microplate reader.

### 4.9. Measurement of Caspase-3/7 Activity

One-step caspase-3/7 assays were performed according to a previously published procedure [84]. For cultures subjected to SAL-induction and Glu-induction, the reaction mixtures (caspase-3,7 buffer and components with cells in 96-multiwell plates) were incubated for 2 h and 3 h at 37 °C, respectively. Caspase-3/7 activity was then measured by an Infinite M200 Pro microplate reader (Tecan, Austria) with 346 and 438 nm excitation and emission wavelengths, respectively.

### 4.10. Statistical Analysis

Experiments were performed in triplicates and repeated in three to five (n = 3–5) independent days. All data are expressed as mean ± SEM. Values for all measured variables are expressed as means ± SEM, which were calculated using Prism 8.4.3 (GraphPad software, La Jolla, CA, USA), which was also used to generate the figures. The statistical analysis was carried by the PAST (ver. 1.97) software package [85]. For differentiation experiment the Student t test was used. The rest of experiments were evaluated by non-parametric Kruskal–Wallis test with Mann–Whitney *post hoc* test with sequential Bonferroni correction. A value of *p* < 0.05 was considered significant.

## Figures and Tables

**Table 1:**
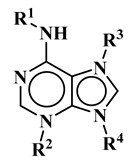
Structures of cytokinins and the positive control agents.

R^1^	R^2^	R^3^	R^4^	Trivial Name	Abbreviation
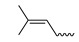	-	-	H	N^6^-isopentenyladenine	iP
	-	-	ribosyl	N^6^-isopentenyladenosine	iPR
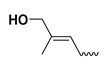	-	-	H	*trans-*zeatin	*t*Z
	-	-	ribosyl	*t-*zeatin riboside	*t*ZR
	-	glycosyl	-	*t-*zeatin-7-glucoside	*t*Z7G
	-	-	glycosyl	*t-*zeatin-9-glucoside	*t*Z9G
	-	-	ribonucleotide	tZR-5‘-monophosphate	*t*ZMP
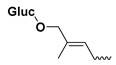	-	-	-	*t-*zeatin-O-glucoside	*t*ZOG
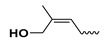	-	-	H	*cis-*zeatin	*c*Z
	-	-	ribosyl	*c-*zeatin riboside	*c*ZR
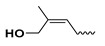	-	-	glycosyl	*c-*zeatin-9-glucoside	cZ9G
	-	-	ribonucleotide	cZR-5‘-monophosphate	cZMP
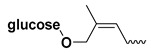	-	-	-	*c-*zeatin-O-glucoside	cZOG
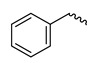	-	-	H	6-benzylaminopurine	BAP
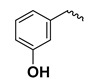	-	-	H	*meta*-topolin	*m*T
	-	-	ribosyl	*meta*-topolin riboside	*m*TR
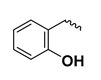	-	-	H	*ortho*-topolin	*o*T
	-	-	ribosyl	*ortho*-topolin riboside	*o*TR
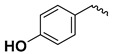	-	-	H	*para*-topolin	*p*T
	-	-	ribosyl	*para*-topolin riboside	*p*TR
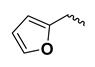	-	-	H	kinetin	K
	-	-	ribosyl	kinetin riboside	KR
	-	-	ribotide	KR-5‘-monophosphate	KMP
	glycosyl	-	-	kinetin-3-glucoside	K3G
	-	-	glycosyl	kinetin-9-glucoside	K9G
	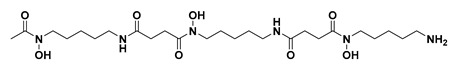				
				Deferoxamine	DFO
	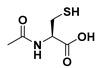			*N*-acetylcysteine	NAC
	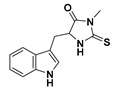			Necrostatin 1	NEC-1

**Table 2:** Oxygen radical absorbance capacity (ORAC) of the tested cytokinins (CKs) expressed as Trolox equivalents (TE) on an equimolar basis. Names, abbreviations, and structures of the CKs are presented in Figure 1.

	Average (TE)	SD (*n* = 3)		Average (TE)	SD (*n* = 3)
*t*Z	0.073	0.012	K	2.082	0.256
*t*ZR	0.13	0.012	KR	1.673	0.294
*t*ZMP	0.326	0.017	KMP	0.726	0.026
*t*Z7G	0.082	0.006	K3G	0.53	0.035
*t*Z9G	0.063	0.004	K9G	0.906	0.025
*t*ZOG	0.042	0.006	BAP	N/A *	N/A *
*c*Z	0.151	0.009	*o*T	7.026	1.179
*c*ZR	0.178	0.005	*o*TR	3.241	0.140
*c*ZMP	0.173	0.036	*m*T	4.509	0.687
*c*Z9G	0.085	0.009	*m*TR	3.171	0.239
*c*ZOG	3.301	0.036	*p*T	16.799	0.829
iP	0.384	0.005	*p*TR	4.147	0.238
iPR	0.224	0.024			

* N/A—no antioxidant capacity was detected.

**Table 3:** Cell viability of neuron-like SH-SY5Y cells after exposure to cytokinins for 24 h. Viability is expressed as percentage of DMSO control.

Compound	Viability *^a^*	Compound	Viability *^a^*
	(%, 10 µM)		(%, 10 µM)
*t*Z	102.3 ± 2.20	K	97.9 ± 1.81
*t*ZR	98.8 ± 1.82	KR	88.1 ± 3.09
*t*ZMP	101.5 ± 4.59	KMP	93.5 ± 3.57
*t*Z7G	101.4 ± 2.44	K3G	99.8 ± 1.13
*t*Z9G	97.6 ± 1.59	K9G	98.3 ± 1.11
*t*ZOG	94.3 ± 1.67	BAP	98.8 ± 1.40
*c*Z	104.0 ± 1.79	*o*T	95.5 ± 4.02
*c*ZR	100.0 ± 1.23	*o*TR	90.8 ± 3.81
*c*ZMP	93.8 ± 2.13	*m*T	90.4 ± 6.03
*c*Z9G	101.7 ± 2.08	*m*TR	99.5 ± 3.69
*c*ZOG	98.18 ± 1.59	*p*T	90.6 ± 3.25
iP	104.5 ± 0.97	*p*TR	89.5 ± 3.89
iPR	96.9 ± 3.25		
NAC (1 mM)	90.2 ± 5.06	DFO (100µM)	102.1 ± 4.22
NEC-1 (50 µM)	95,8 ± 3.75		

*^a^* viabilities are expressed as means ± SEM, compounds were tested in three independent experiments in triplicates.

## Data Availability

Data is contained within the article or supplementary material.

## Supplementary Materials

Original images are available online.

## Sample Availability

Samples of the compounds are not available from the authors.
