# Supplementary figures and images for: Cytokinin Plant Hormones Have Neuroprotective Activity in In Vitro Models of Parkinson’s Disease
Source: Molecules. 2021 Jan 12;26(2):361. doi: 10.3390/molecules26020361 (PMC7827283; doi:10.3390/molecules26020361)

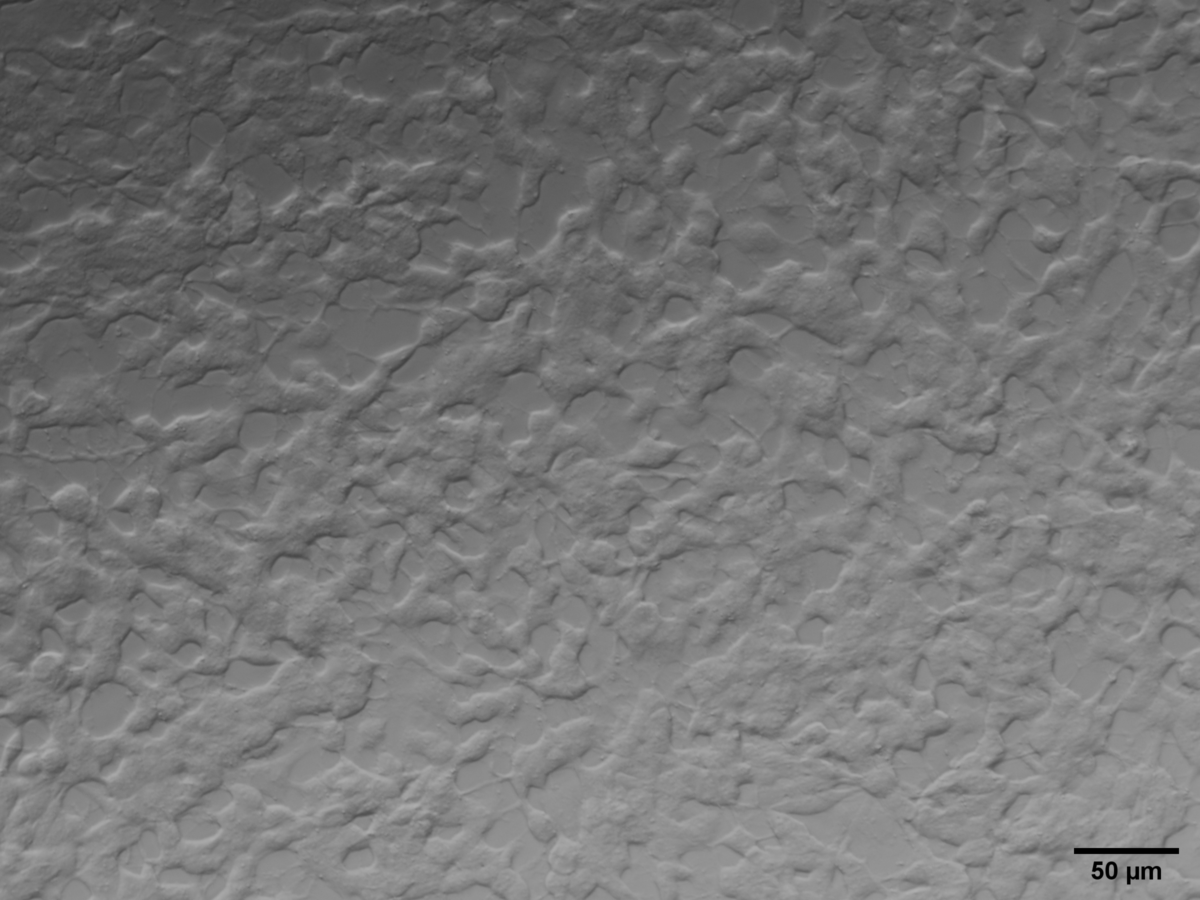

Supplement: Supplementary file 1 [file molecules-26-00361-s001.zip › images_for submission/DHE_Glutamate/Brightfield/CTR_BF.tif]

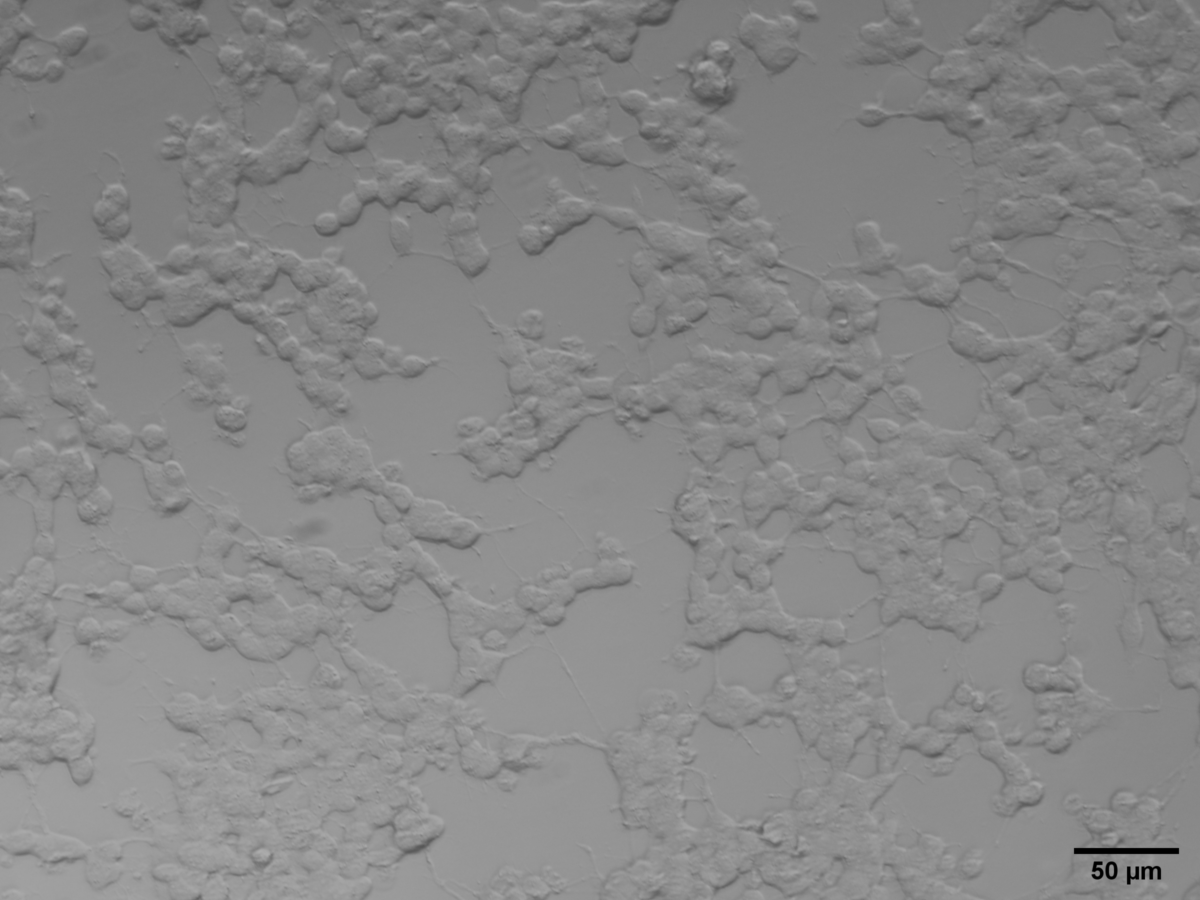

Supplement: Supplementary file 1 [file molecules-26-00361-s001.zip › images_for submission/DHE_Glutamate/Brightfield/cZ_BF.tif]

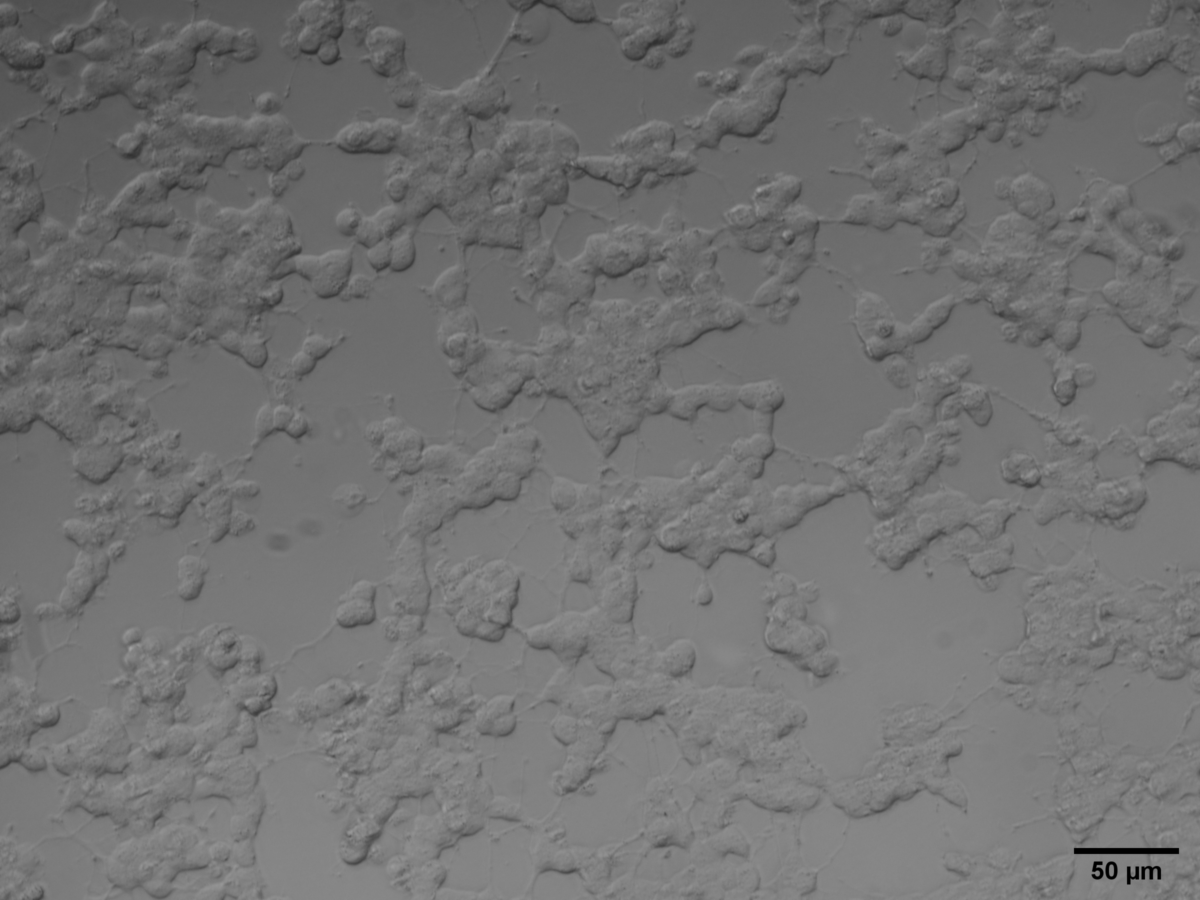

Supplement: Supplementary file 1 [file molecules-26-00361-s001.zip › images_for submission/DHE_Glutamate/Brightfield/DFO.tif]

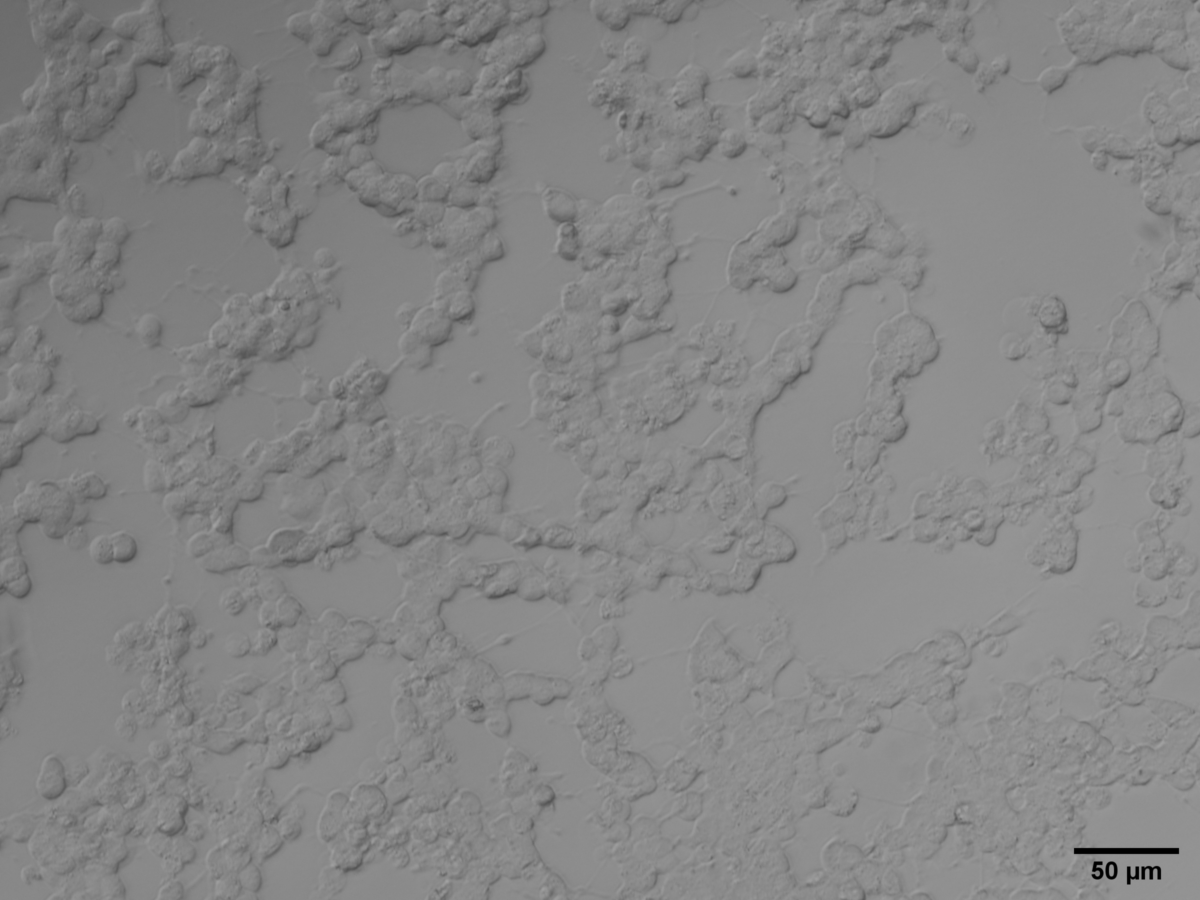

Supplement: Supplementary file 1 [file molecules-26-00361-s001.zip › images_for submission/DHE_Glutamate/Brightfield/glu_BF.tif]

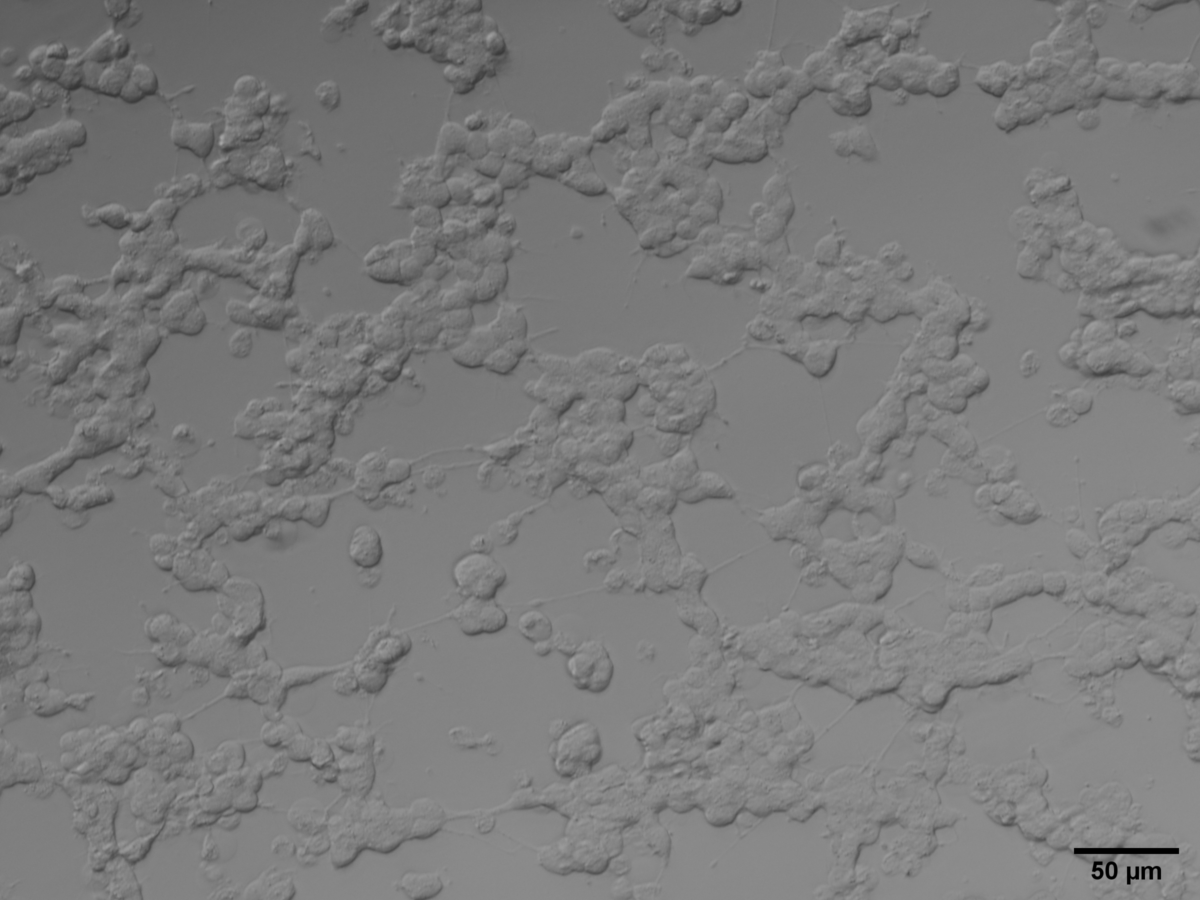

Supplement: Supplementary file 1 [file molecules-26-00361-s001.zip › images_for submission/DHE_Glutamate/Brightfield/NEC_1_BF.tif]

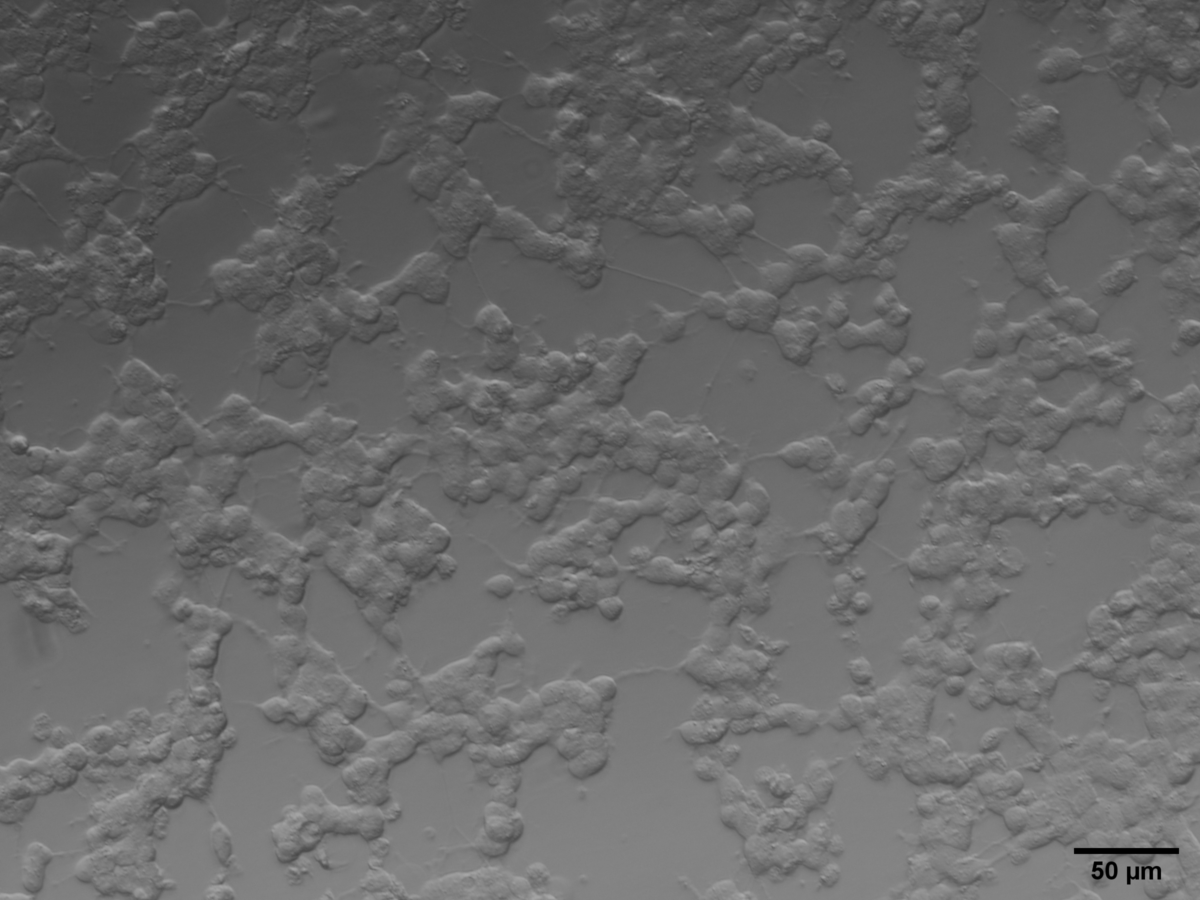

Supplement: Supplementary file 1 [file molecules-26-00361-s001.zip › images_for submission/DHE_Glutamate/Brightfield/tZ_BF.tif]

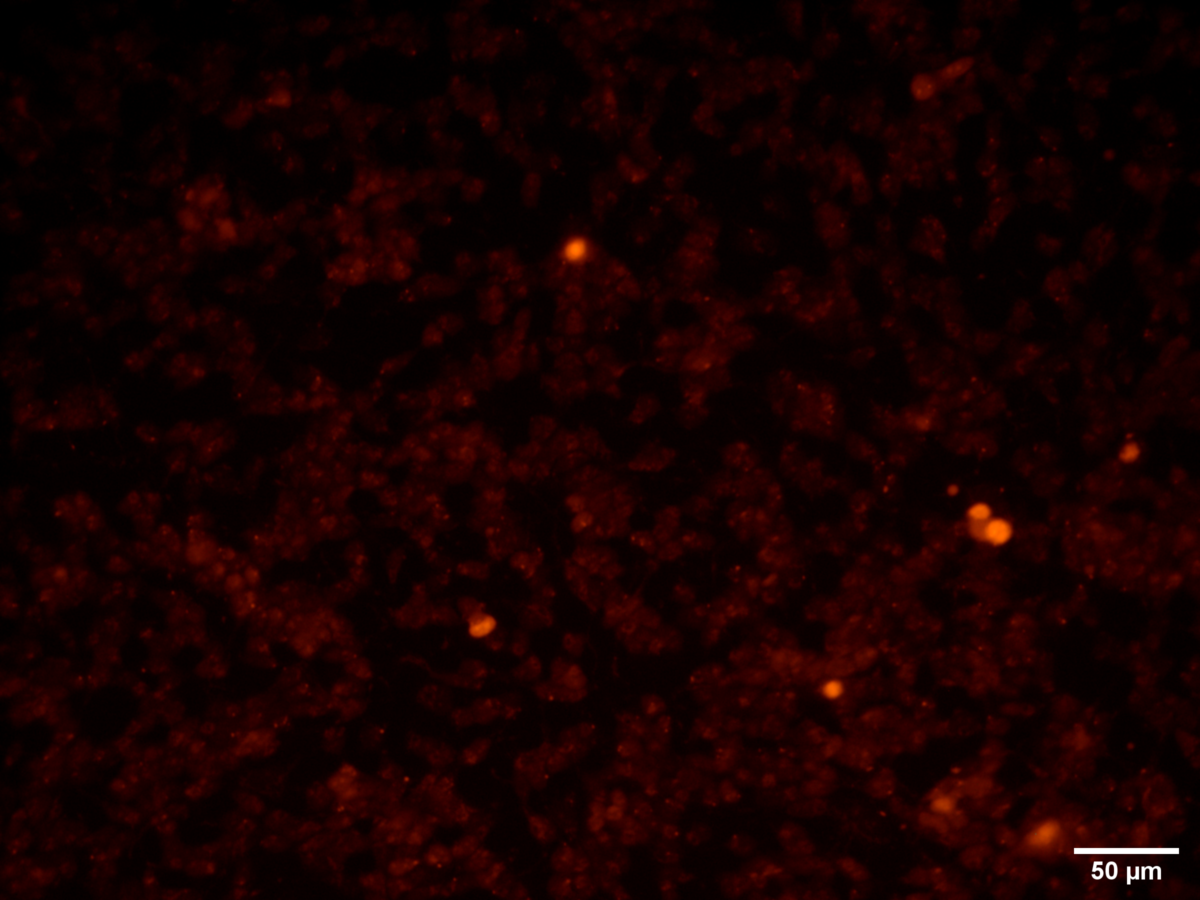

Supplement: Supplementary file 1 [file molecules-26-00361-s001.zip › images_for submission/DHE_Glutamate/DHE staining adjusted/CTR_DHE.tif]

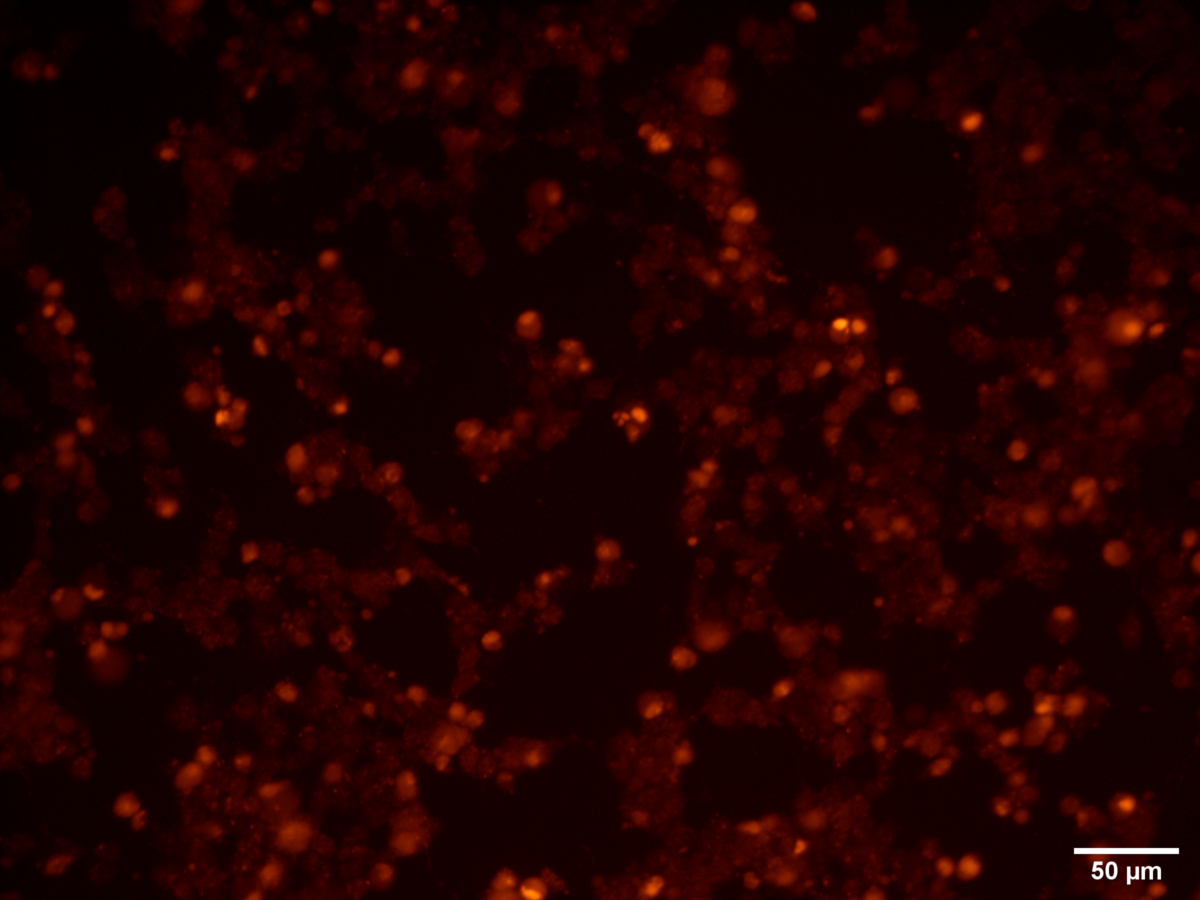

Supplement: Supplementary file 1 [file molecules-26-00361-s001.zip › images_for submission/DHE_Glutamate/DHE staining adjusted/cZ_DHE.tif]

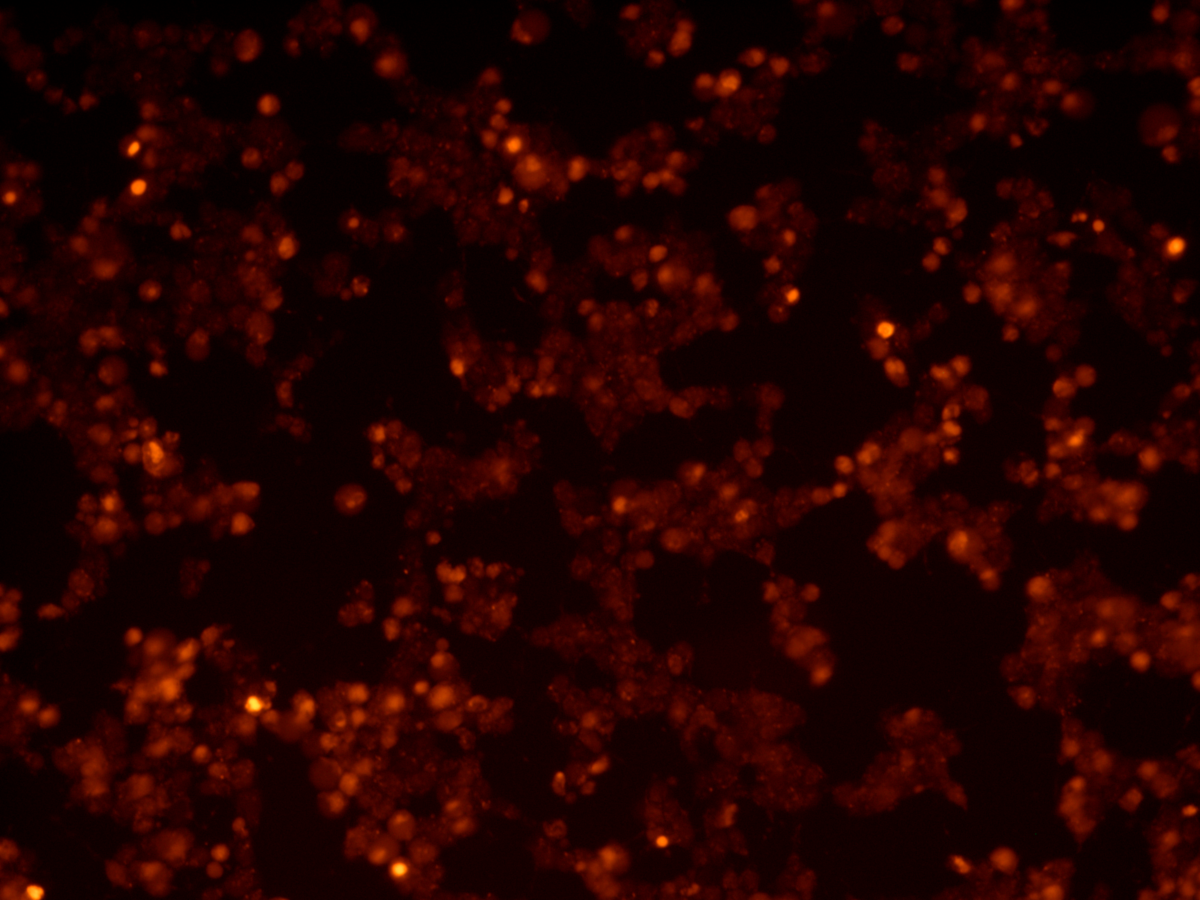

Supplement: Supplementary file 1 [file molecules-26-00361-s001.zip › images_for submission/DHE_Glutamate/DHE staining adjusted/DFO_DHE.tif]

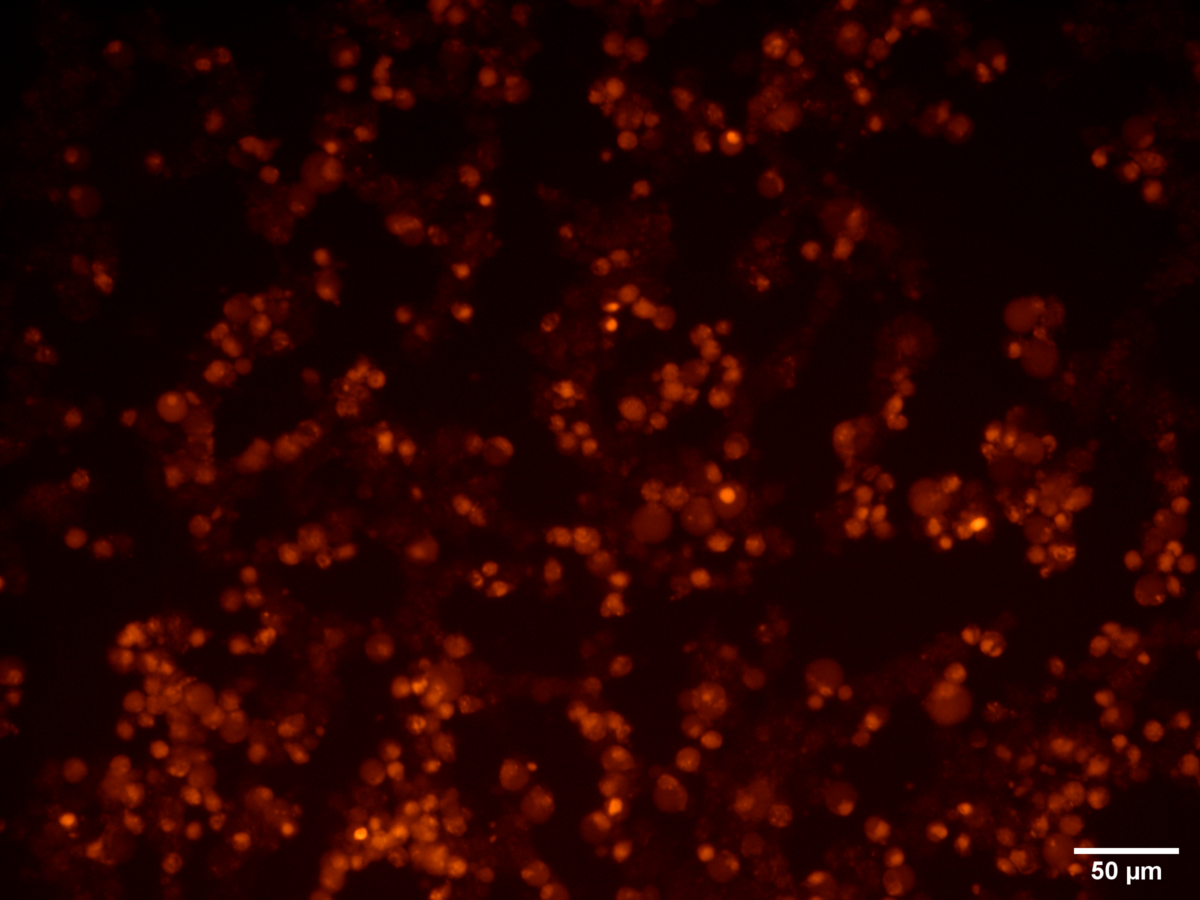

Supplement: Supplementary file 1 [file molecules-26-00361-s001.zip › images_for submission/DHE_Glutamate/DHE staining adjusted/glu_DHE.tif]

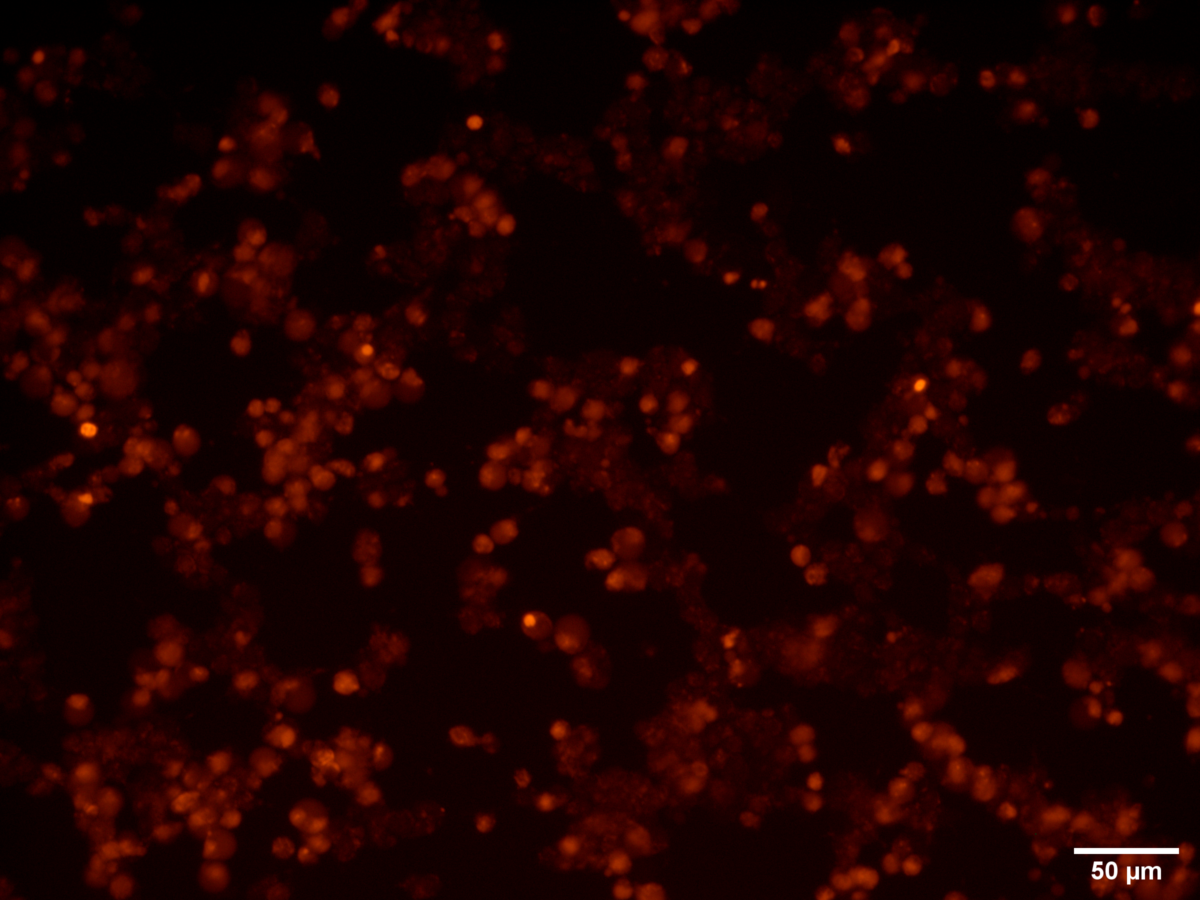

Supplement: Supplementary file 1 [file molecules-26-00361-s001.zip › images_for submission/DHE_Glutamate/DHE staining adjusted/NEC_1_DHE.tif]

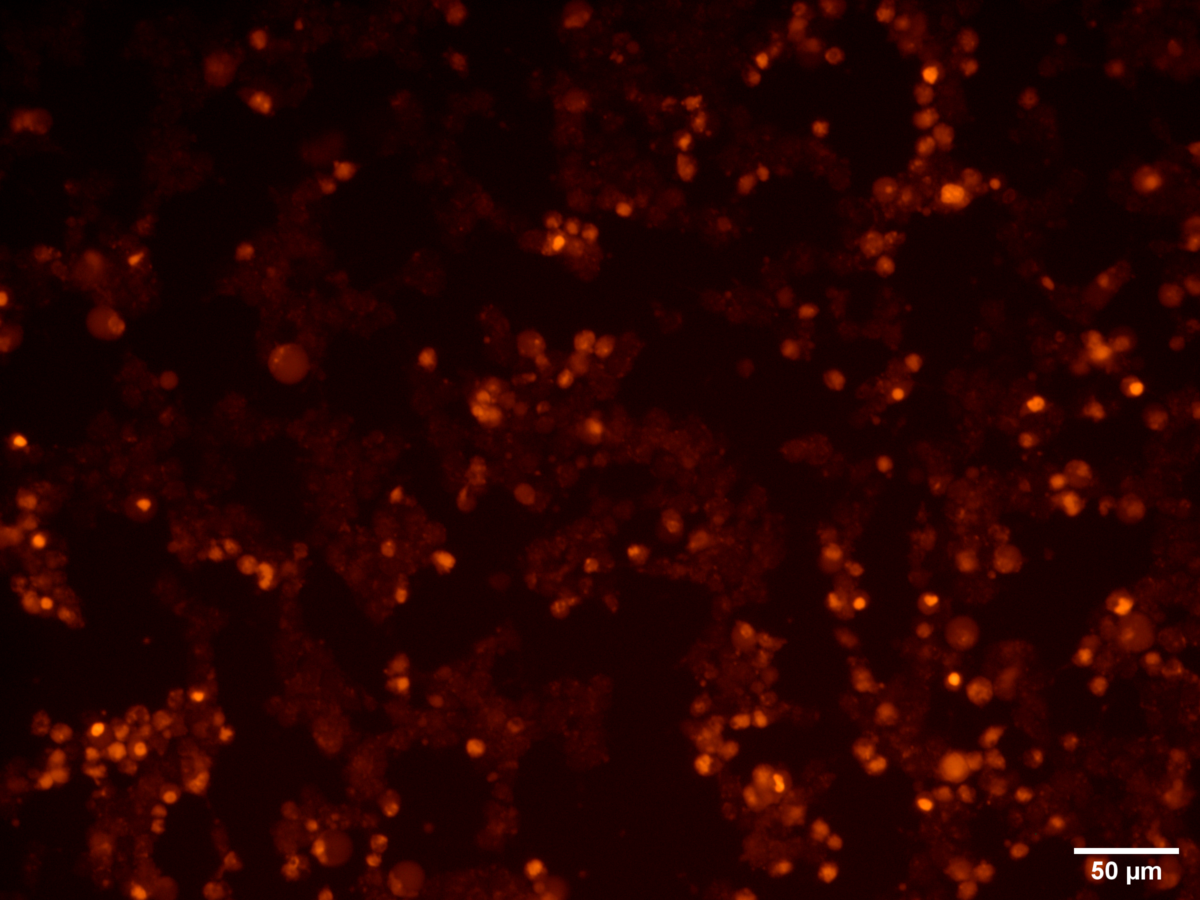

Supplement: Supplementary file 1 [file molecules-26-00361-s001.zip › images_for submission/DHE_Glutamate/DHE staining adjusted/tZ_DHE.tif]

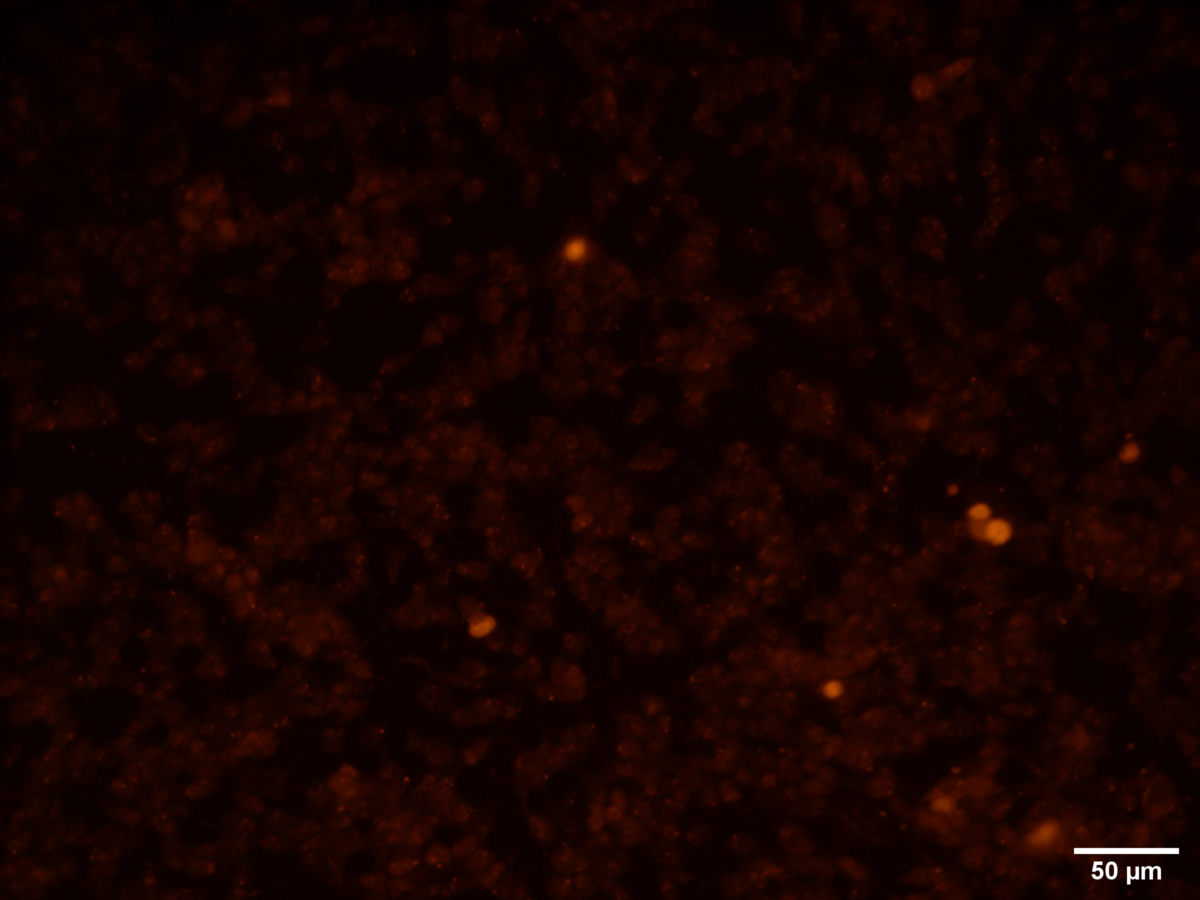

Supplement: Supplementary file 1 [file molecules-26-00361-s001.zip › images_for submission/DHE_Glutamate/DHE staining original/CTR_DHE.tif]

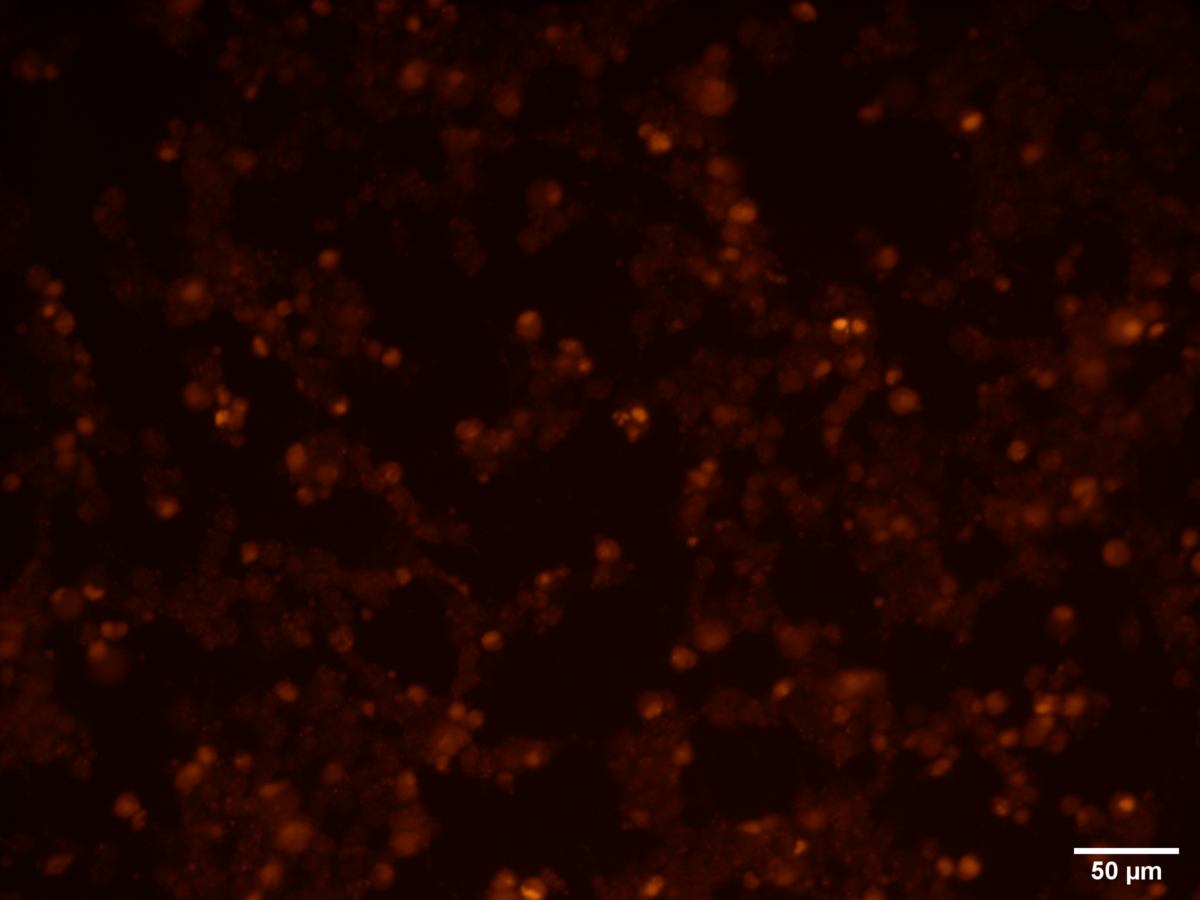

Supplement: Supplementary file 1 [file molecules-26-00361-s001.zip › images_for submission/DHE_Glutamate/DHE staining original/cZ_DHE.tif]

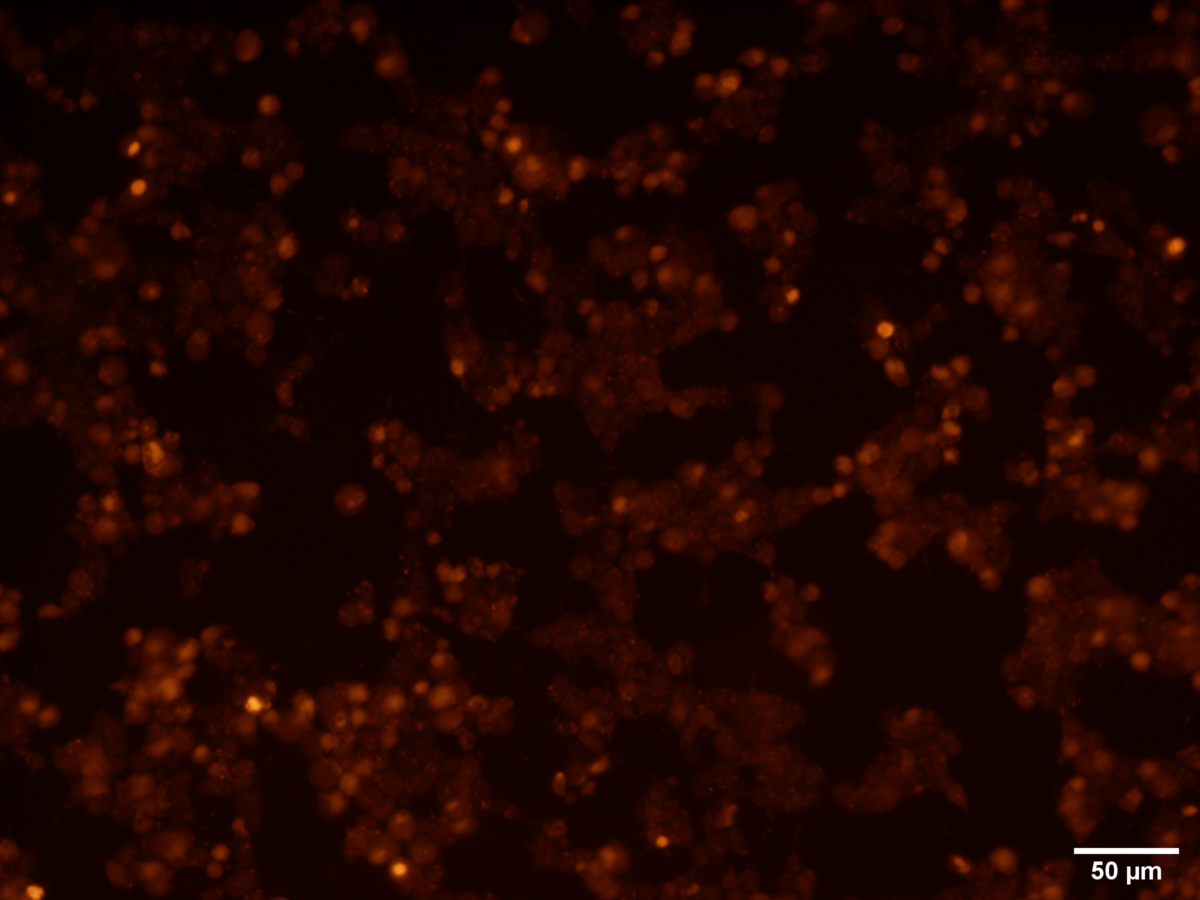

Supplement: Supplementary file 1 [file molecules-26-00361-s001.zip › images_for submission/DHE_Glutamate/DHE staining original/DFO_DHE.tif]

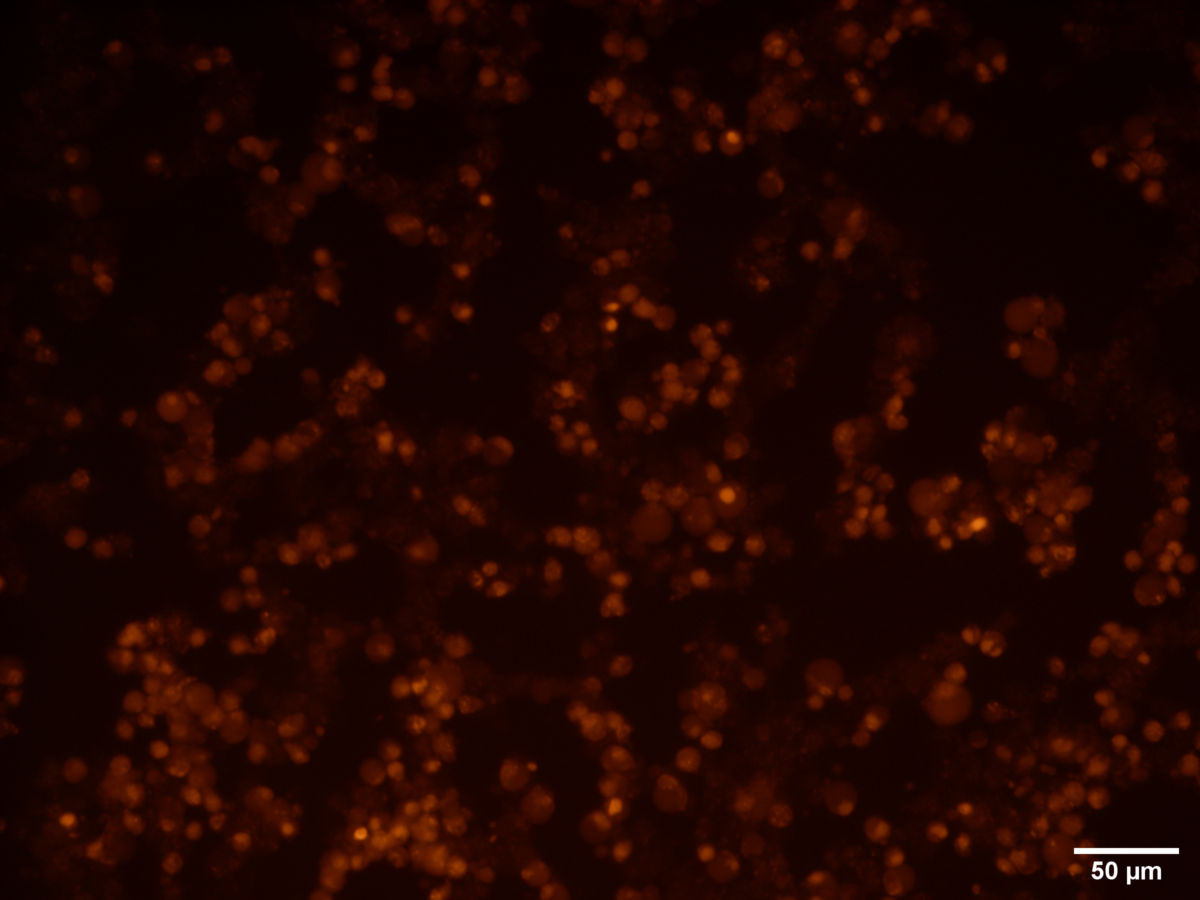

Supplement: Supplementary file 1 [file molecules-26-00361-s001.zip › images_for submission/DHE_Glutamate/DHE staining original/glu_DHE.tif]

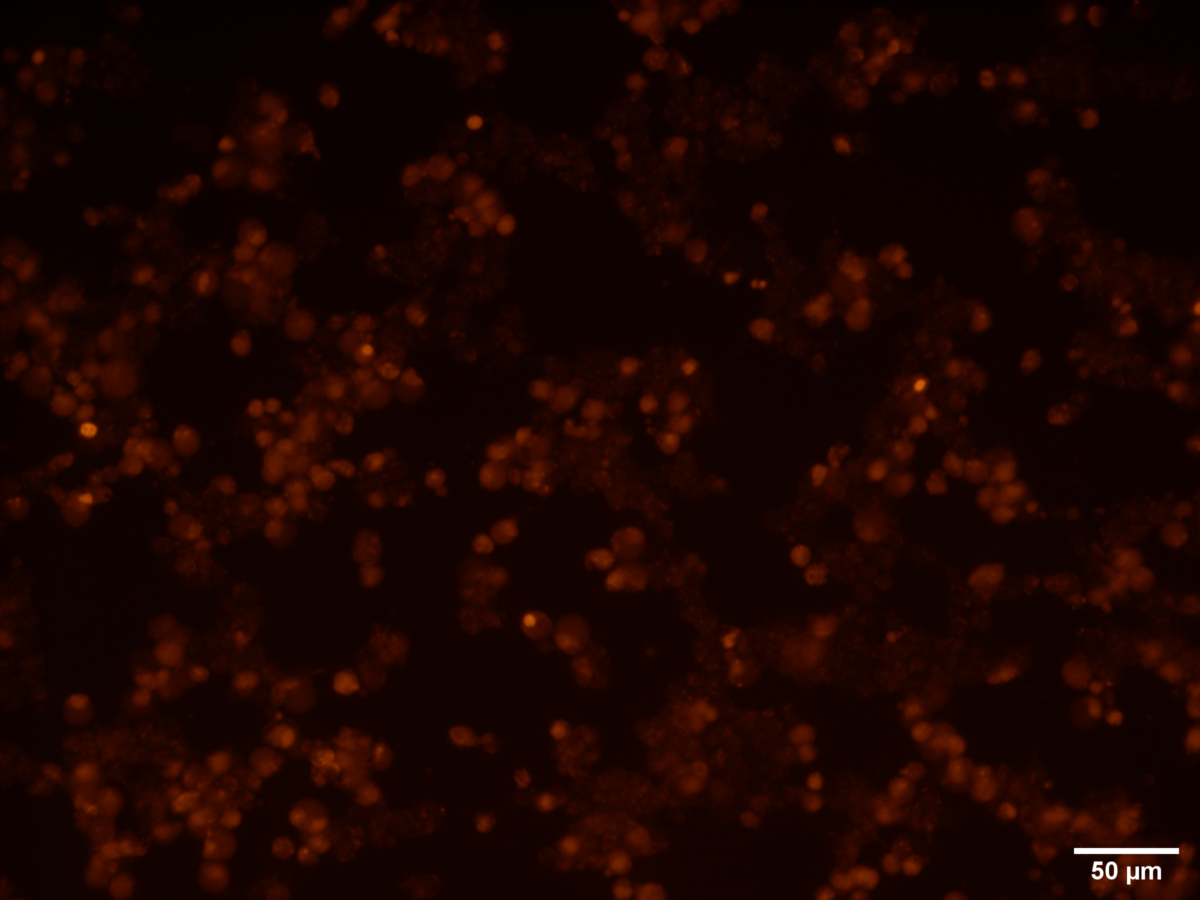

Supplement: Supplementary file 1 [file molecules-26-00361-s001.zip › images_for submission/DHE_Glutamate/DHE staining original/NEC_1_DHE.tif]

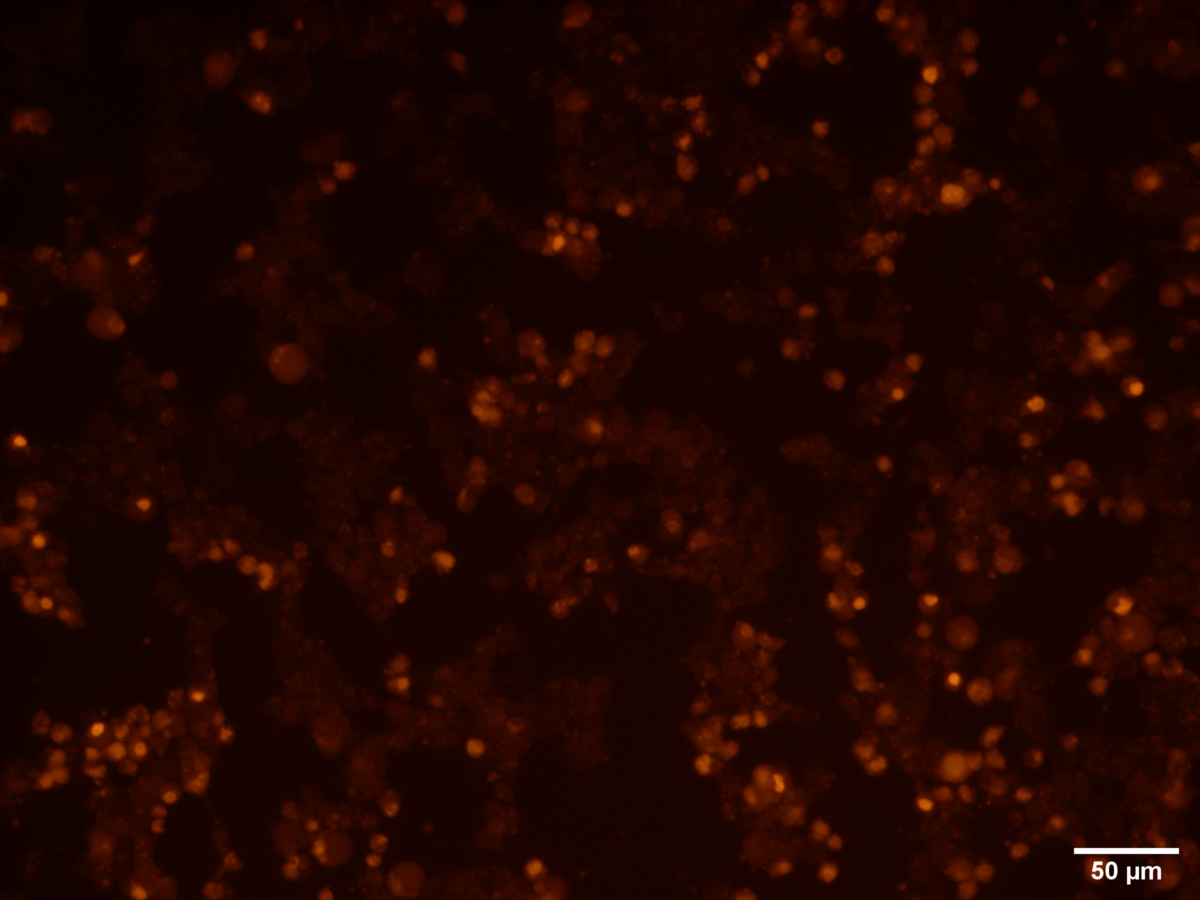

Supplement: Supplementary file 1 [file molecules-26-00361-s001.zip › images_for submission/DHE_Glutamate/DHE staining original/tZ_DHE.tif]

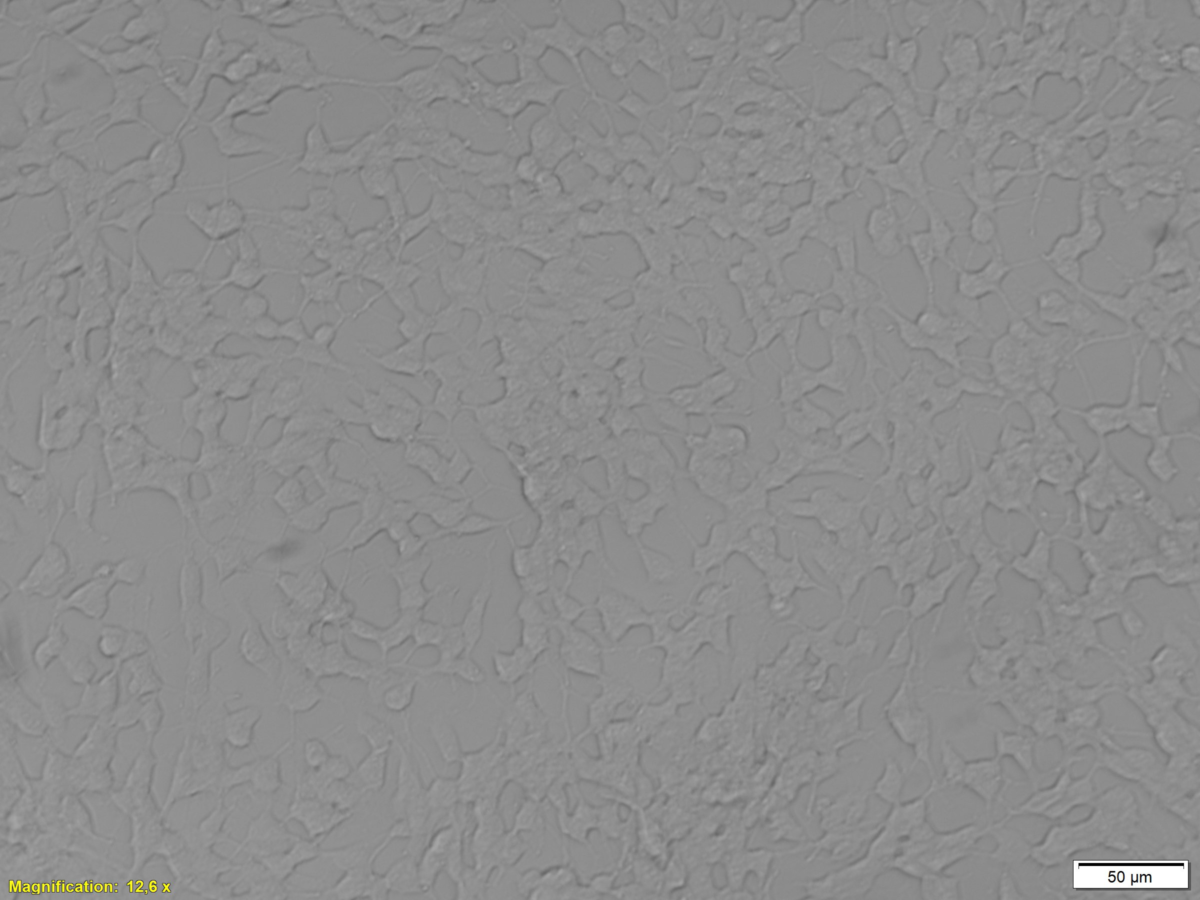

Supplement: Supplementary file 1 [file molecules-26-00361-s001.zip › images_for submission/DHE_Salsolinol/Brightfield/CTR_BF.tif]

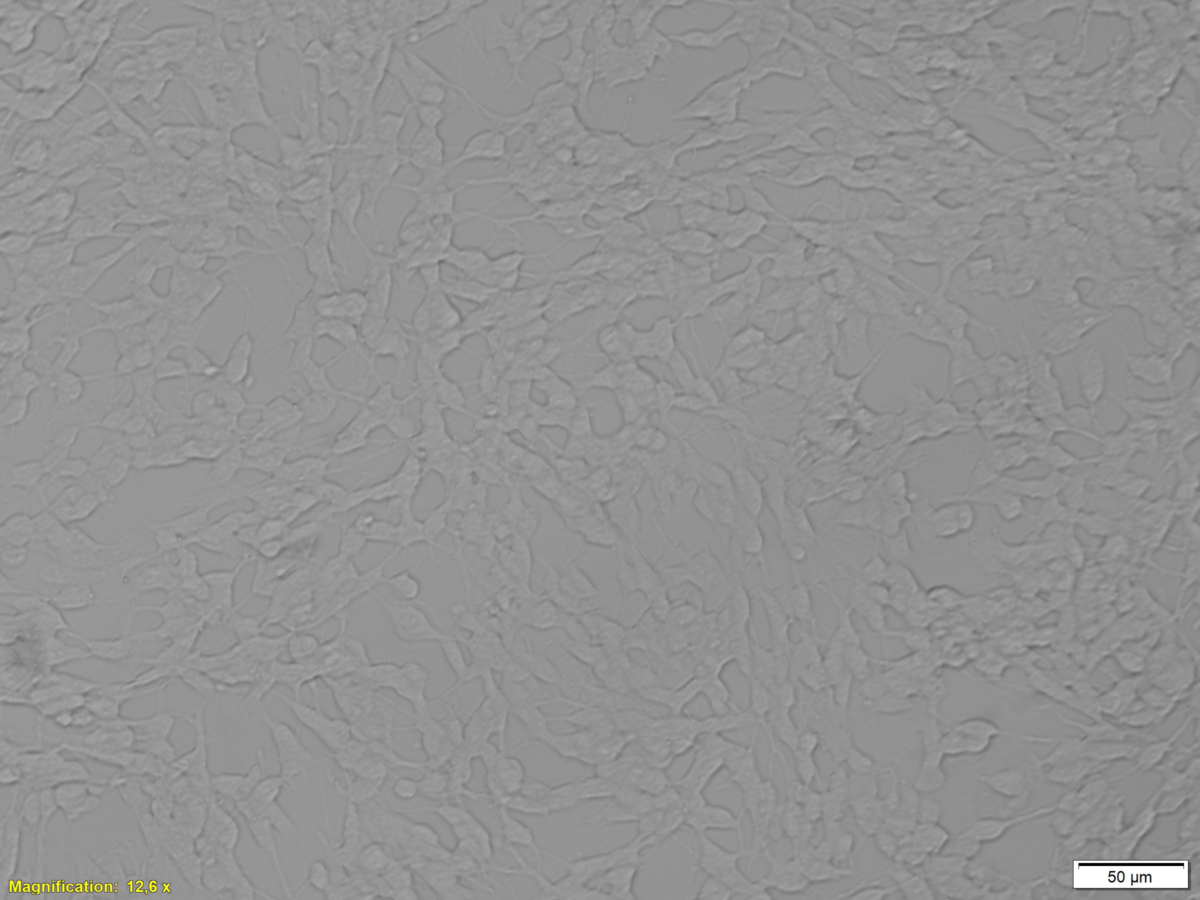

Supplement: Supplementary file 1 [file molecules-26-00361-s001.zip › images_for submission/DHE_Salsolinol/Brightfield/cZR_BF.tif]

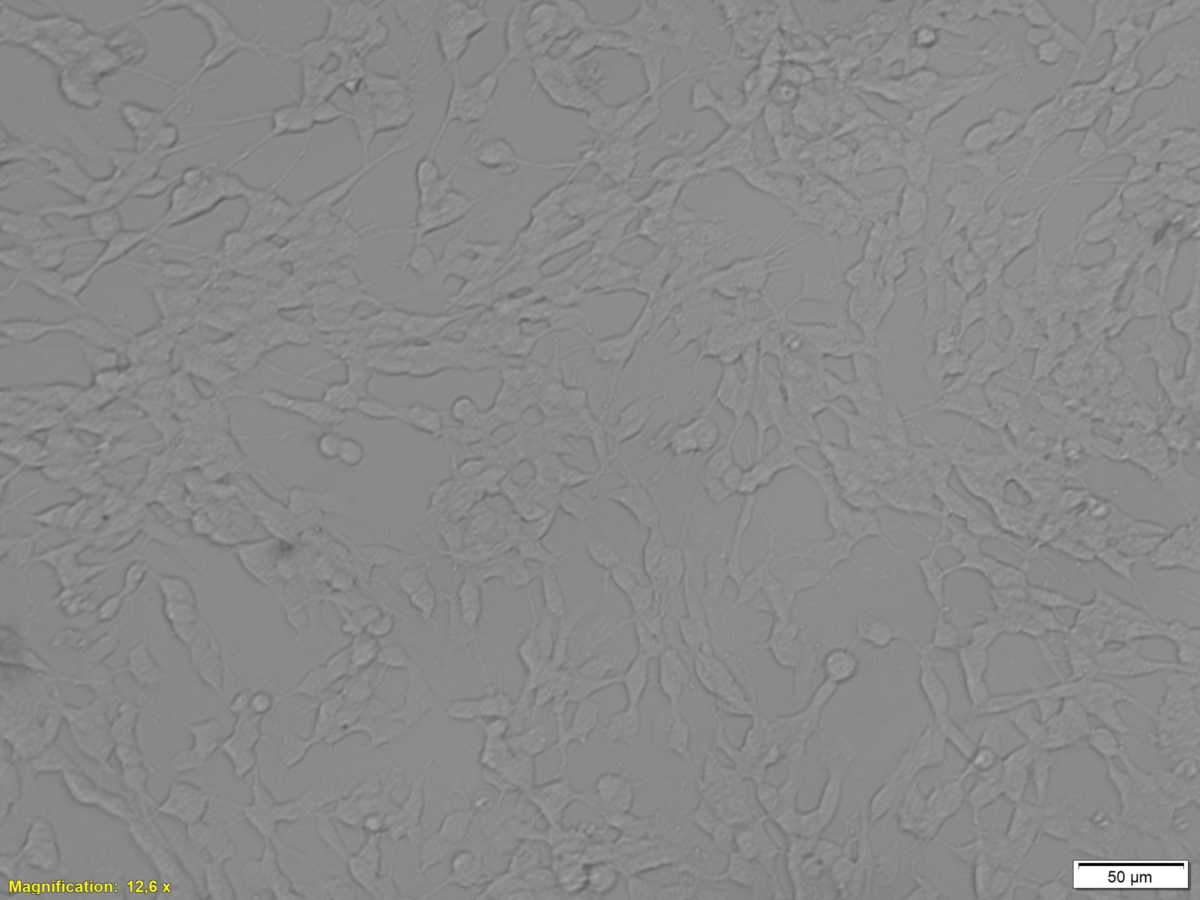

Supplement: Supplementary file 1 [file molecules-26-00361-s001.zip › images_for submission/DHE_Salsolinol/Brightfield/IPR_BF.tif]

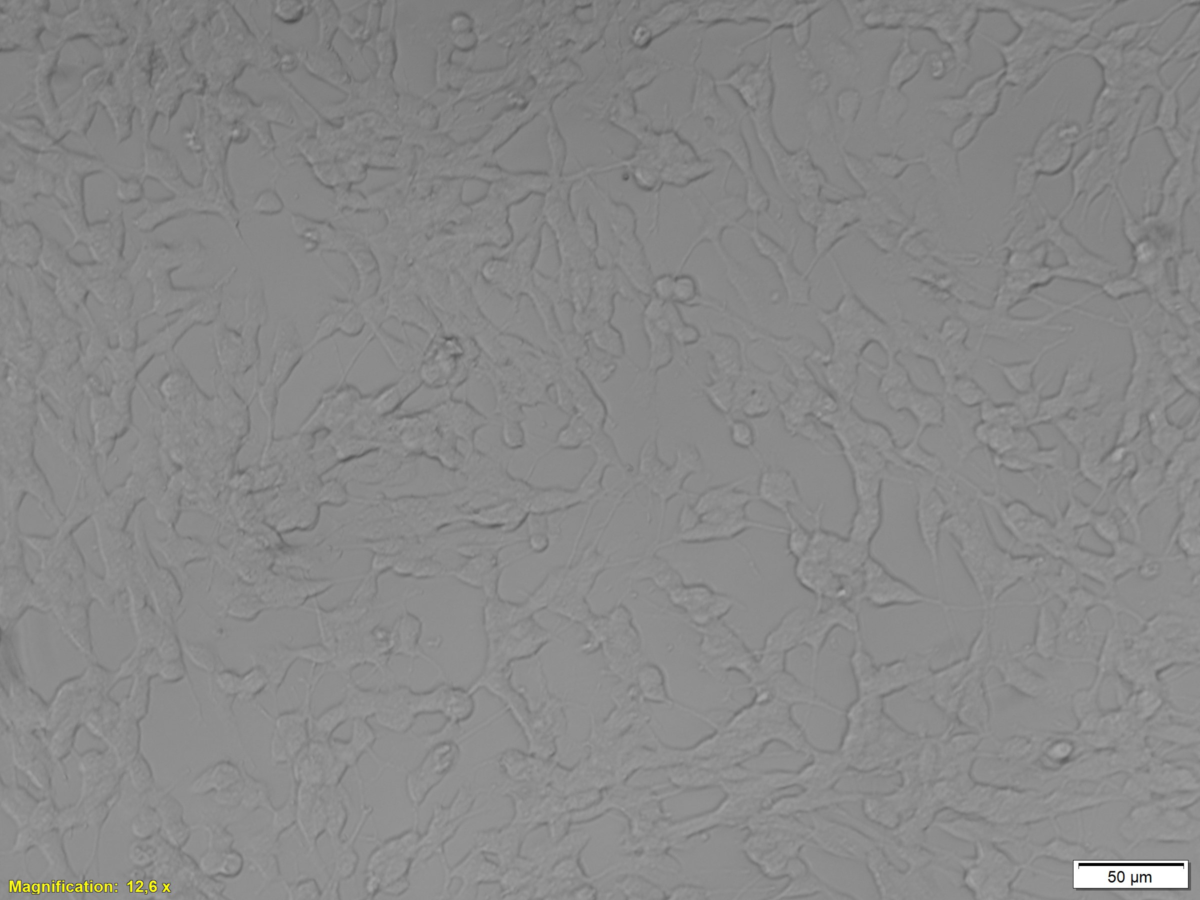

Supplement: Supplementary file 1 [file molecules-26-00361-s001.zip › images_for submission/DHE_Salsolinol/Brightfield/K3G_BF.tif]

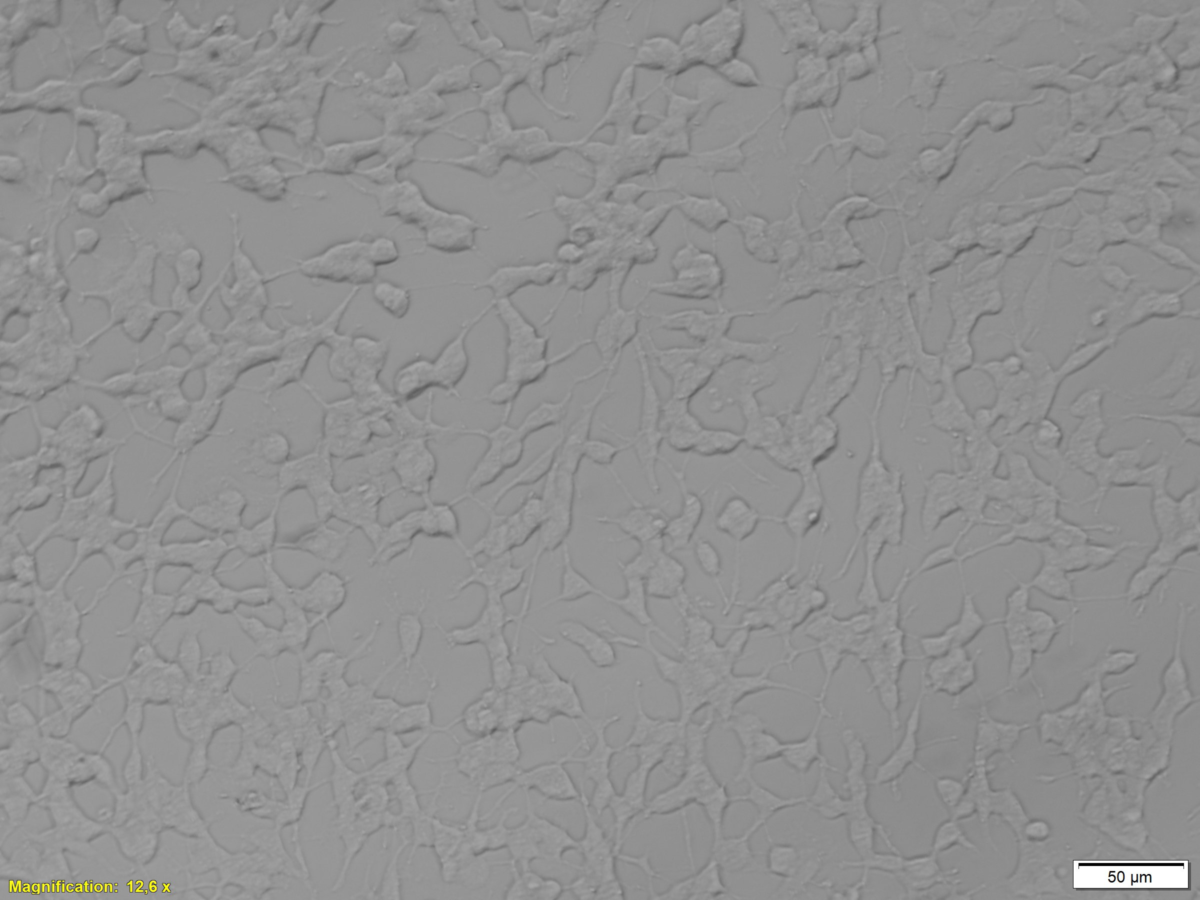

Supplement: Supplementary file 1 [file molecules-26-00361-s001.zip › images_for submission/DHE_Salsolinol/Brightfield/NAC_BF.tif]

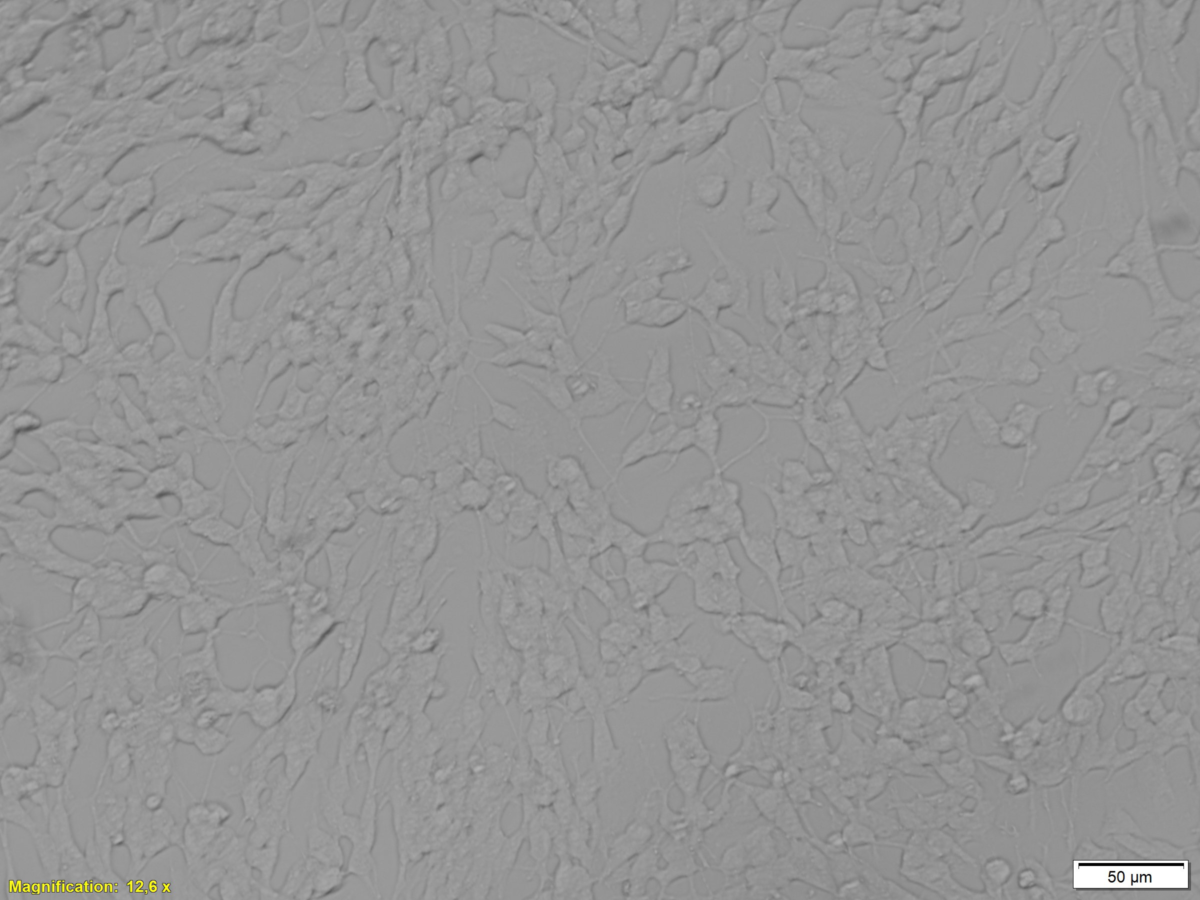

Supplement: Supplementary file 1 [file molecules-26-00361-s001.zip › images_for submission/DHE_Salsolinol/Brightfield/SAL_BF.tif]

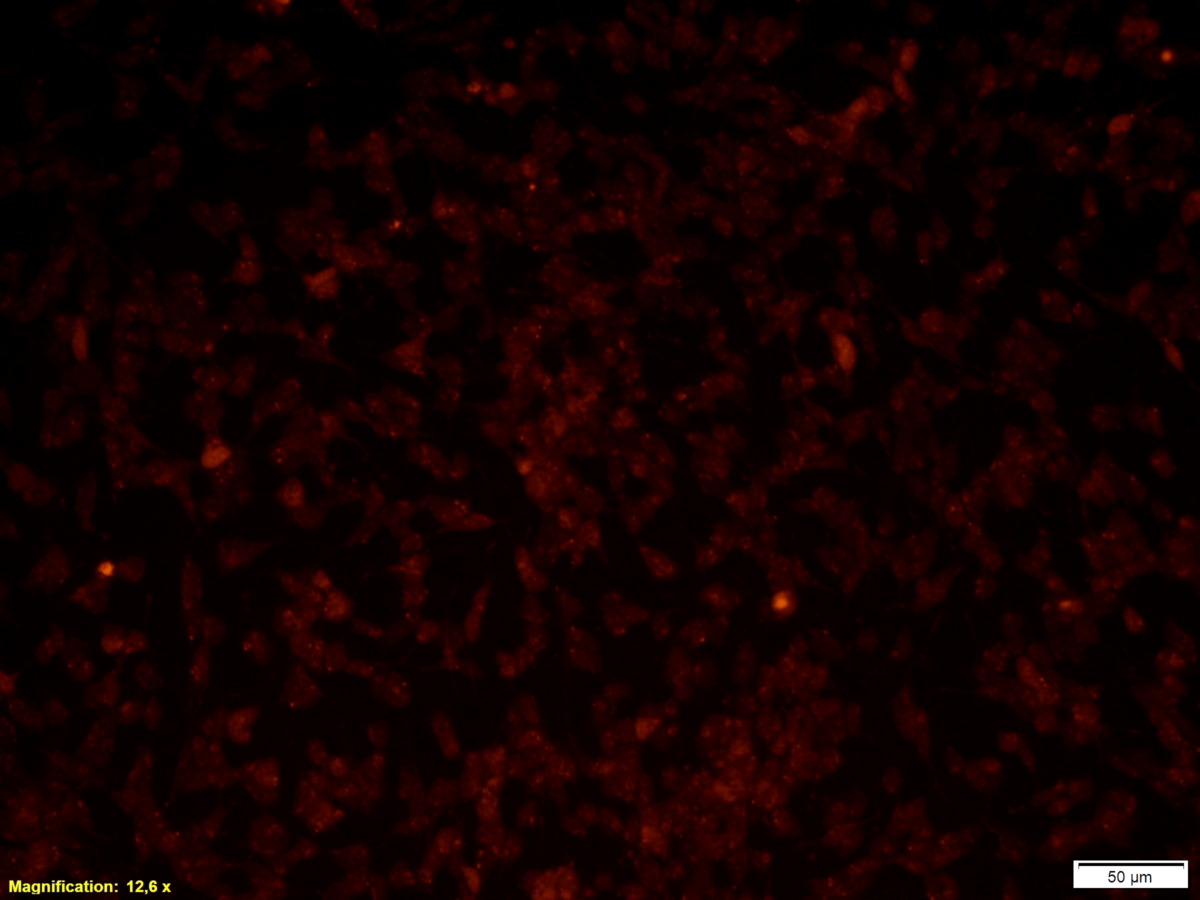

Supplement: Supplementary file 1 [file molecules-26-00361-s001.zip › images_for submission/DHE_Salsolinol/DHE_Salsolinol_adjusted/CTR_DHE.tif]

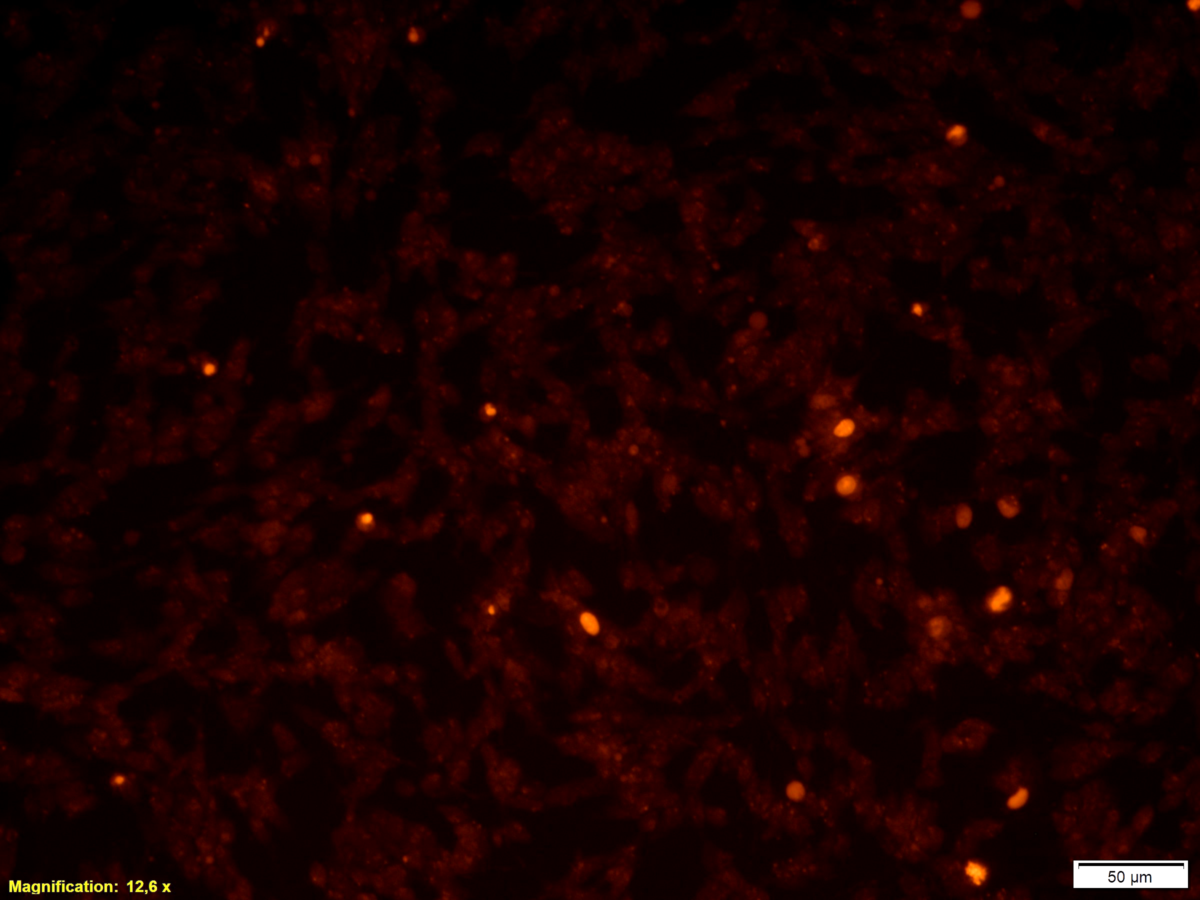

Supplement: Supplementary file 1 [file molecules-26-00361-s001.zip › images_for submission/DHE_Salsolinol/DHE_Salsolinol_adjusted/cZR_DHE.tif]

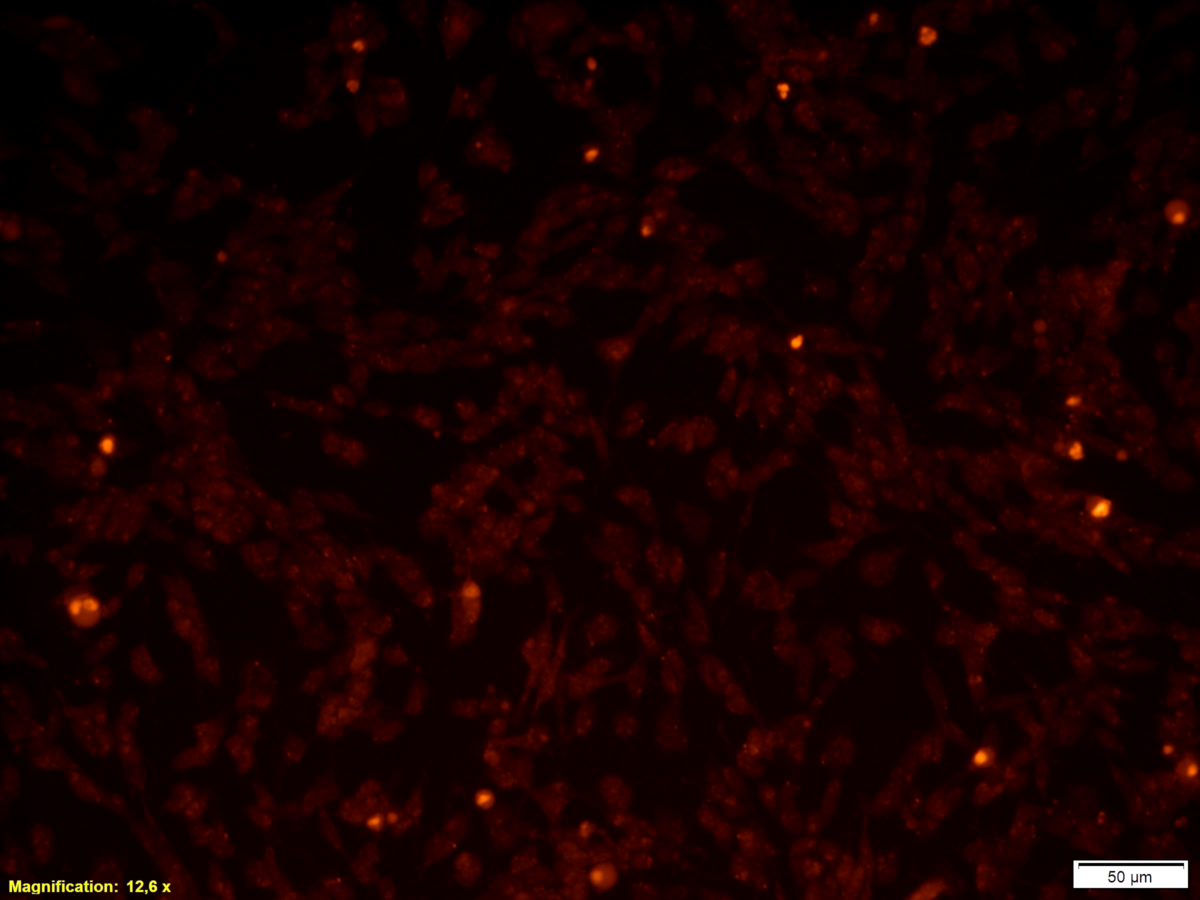

Supplement: Supplementary file 1 [file molecules-26-00361-s001.zip › images_for submission/DHE_Salsolinol/DHE_Salsolinol_adjusted/IPR_DHE.tif]

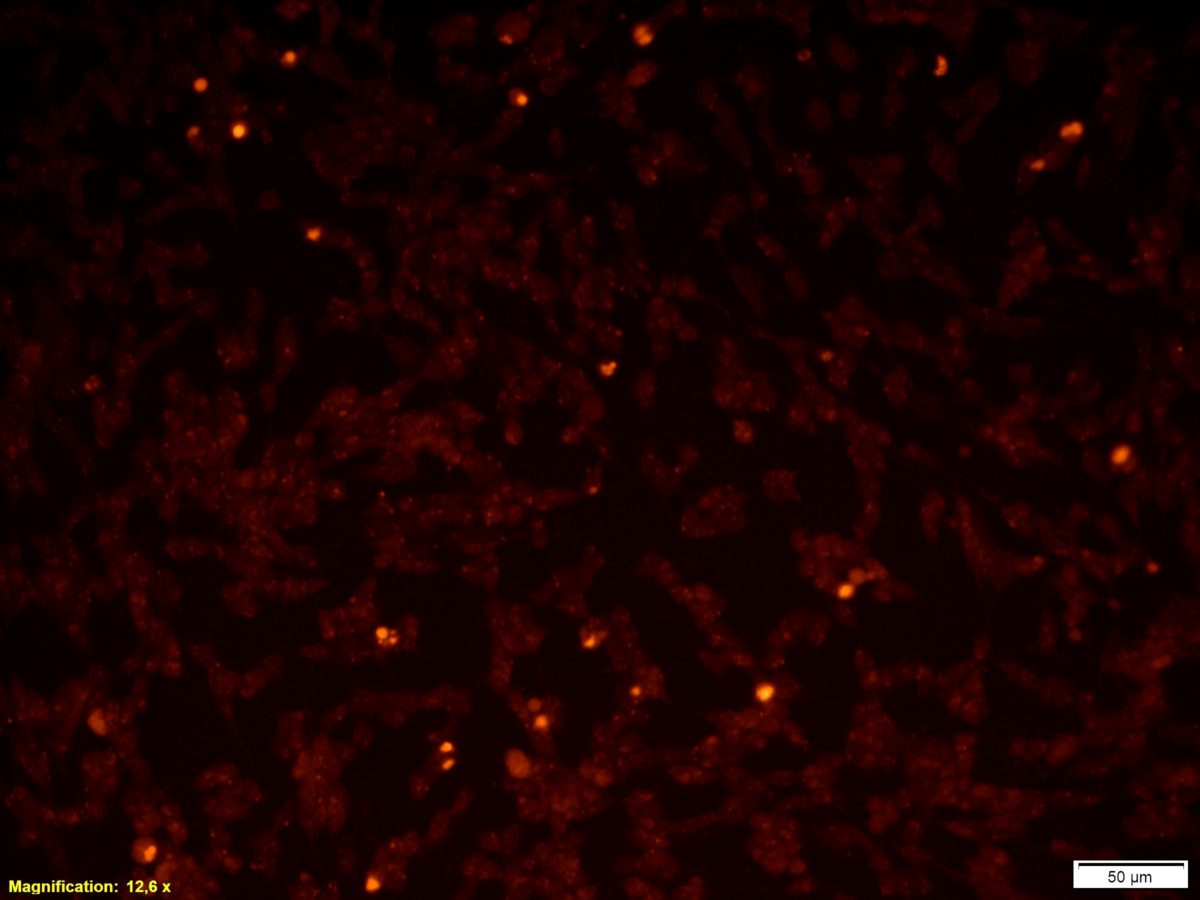

Supplement: Supplementary file 1 [file molecules-26-00361-s001.zip › images_for submission/DHE_Salsolinol/DHE_Salsolinol_adjusted/K3G_DHE.tif]

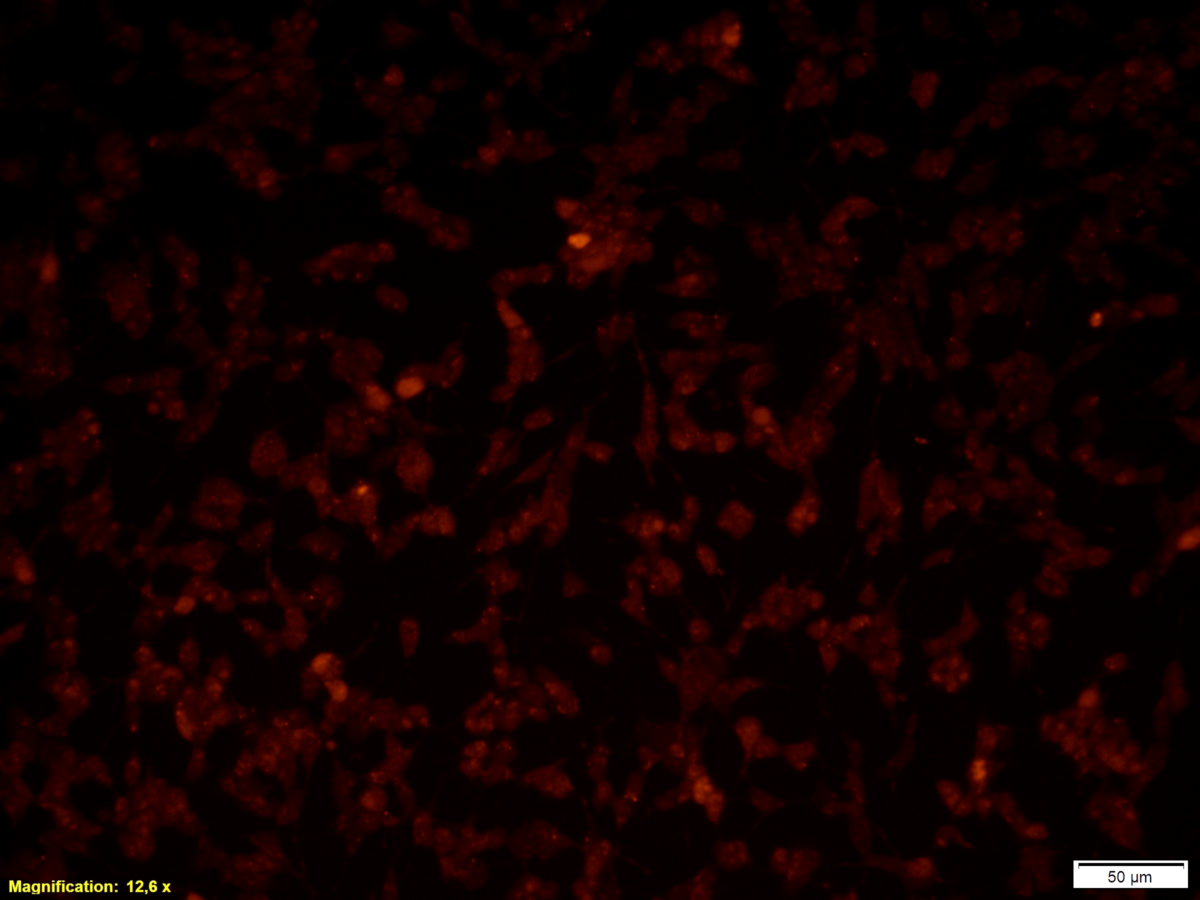

Supplement: Supplementary file 1 [file molecules-26-00361-s001.zip › images_for submission/DHE_Salsolinol/DHE_Salsolinol_adjusted/NAC_DHE.tif]

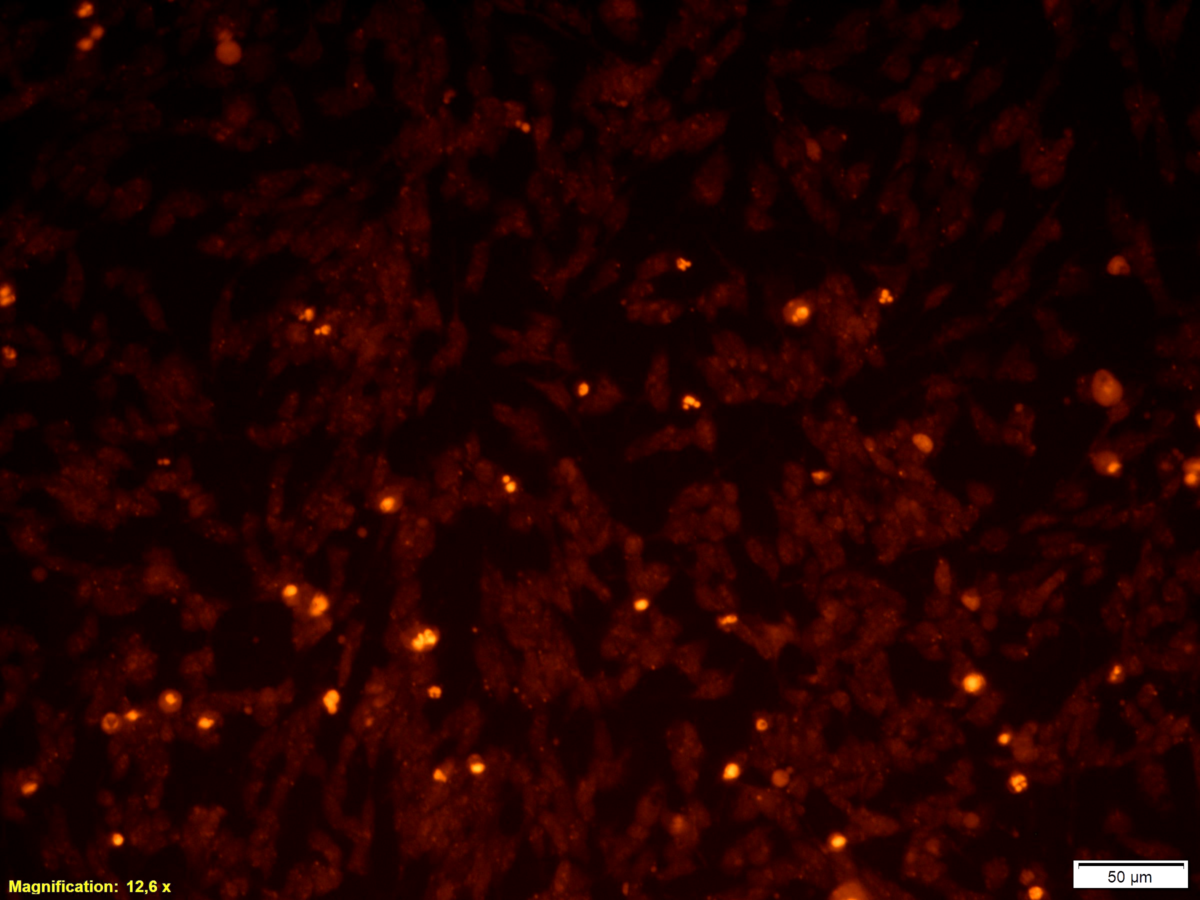

Supplement: Supplementary file 1 [file molecules-26-00361-s001.zip › images_for submission/DHE_Salsolinol/DHE_Salsolinol_adjusted/SAL_DHE-.tif]

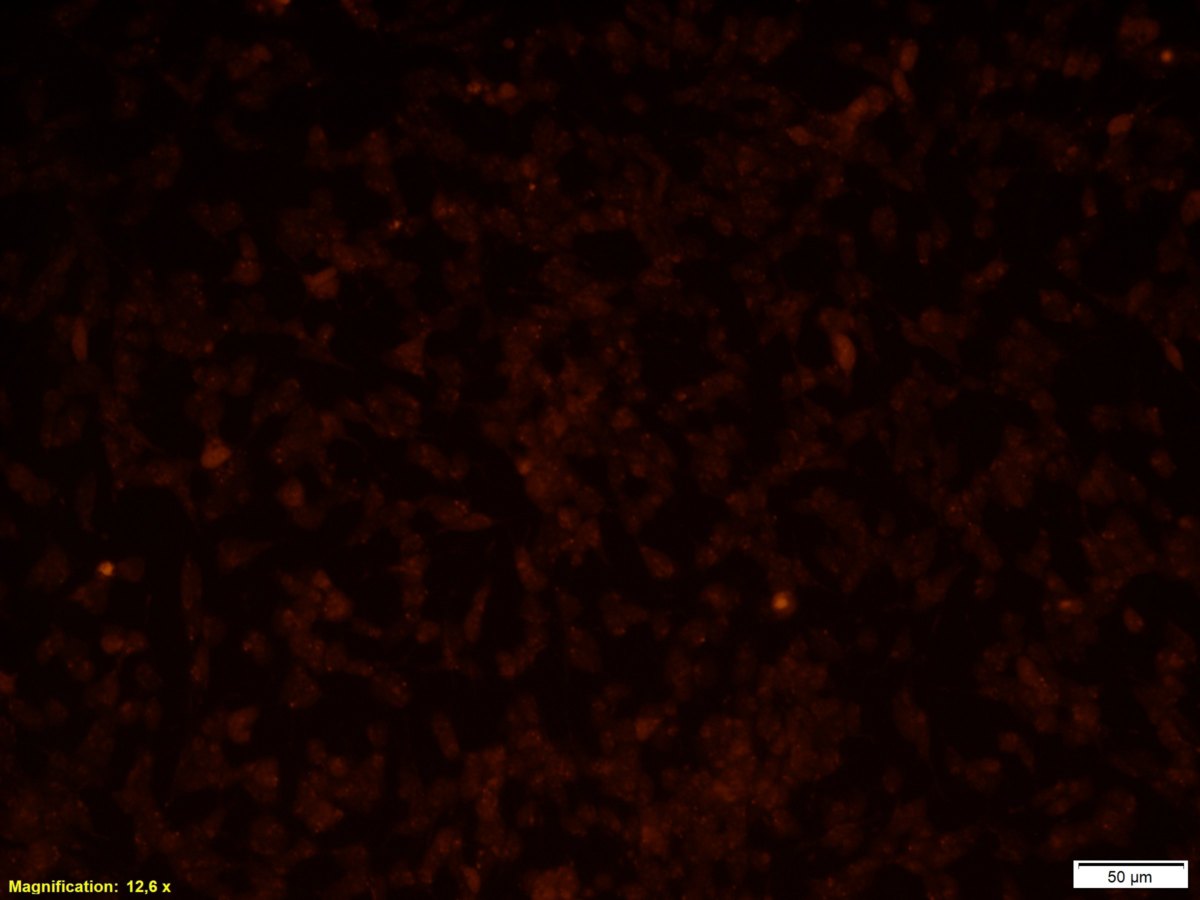

Supplement: Supplementary file 1 [file molecules-26-00361-s001.zip › images_for submission/DHE_Salsolinol/DHE_Salsolinol_original/CTR_DHE.tif]

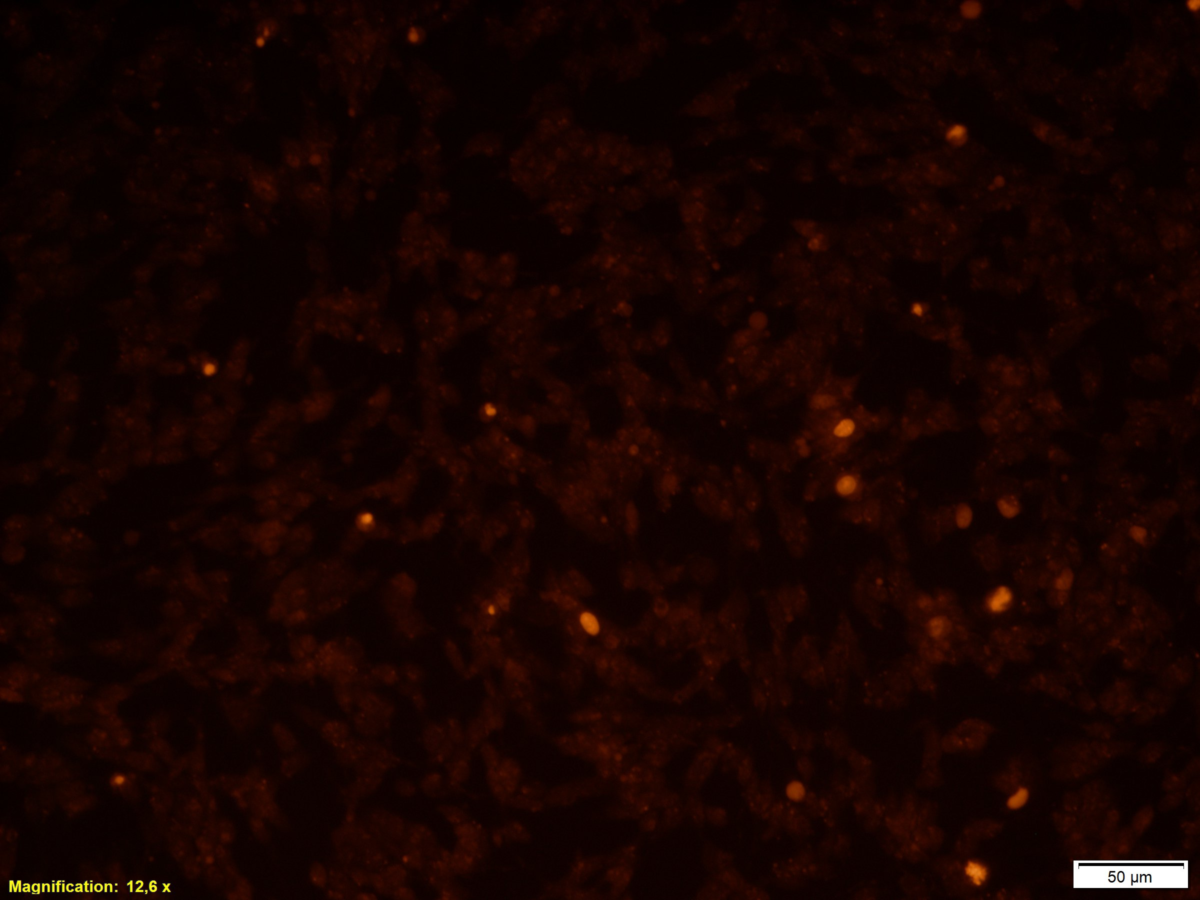

Supplement: Supplementary file 1 [file molecules-26-00361-s001.zip › images_for submission/DHE_Salsolinol/DHE_Salsolinol_original/cZR_DHE.tif]

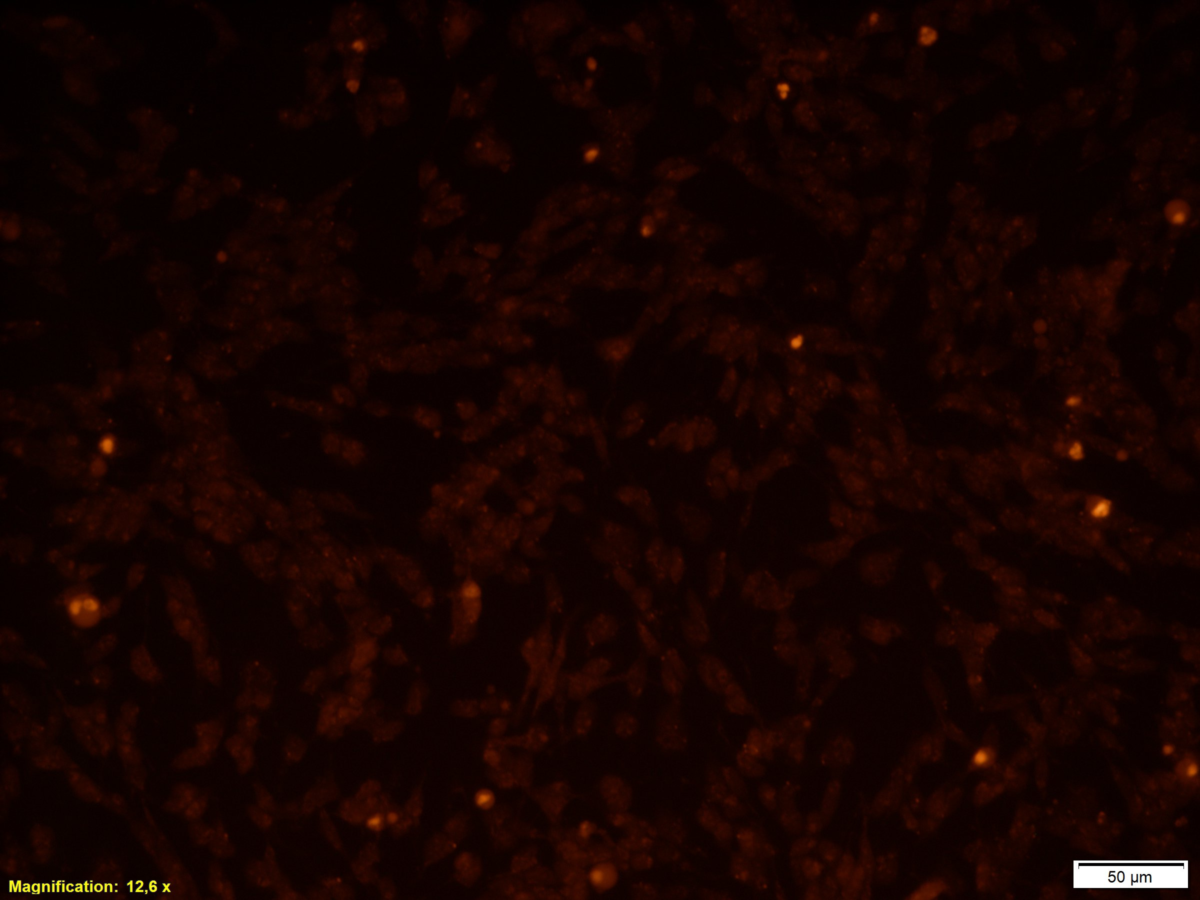

Supplement: Supplementary file 1 [file molecules-26-00361-s001.zip › images_for submission/DHE_Salsolinol/DHE_Salsolinol_original/IPR_DHE.tif]

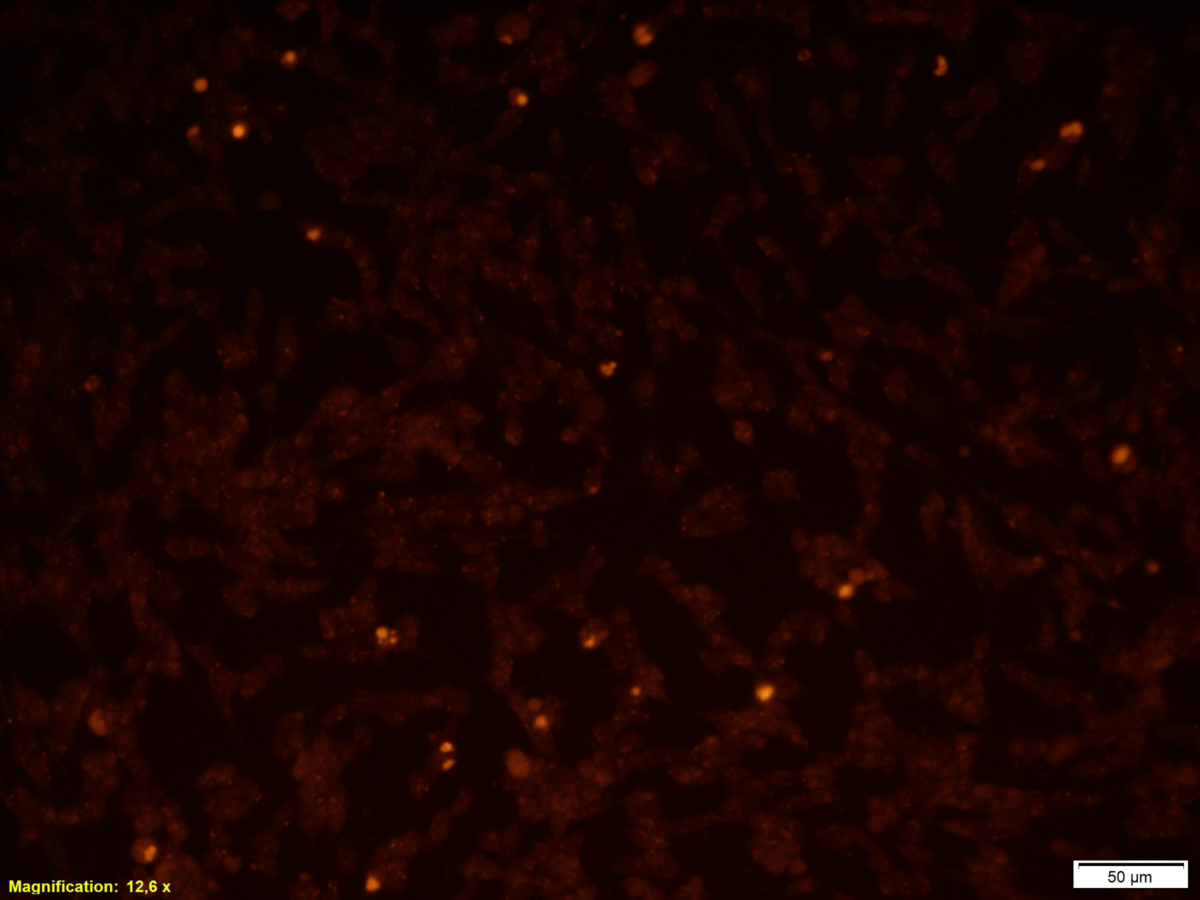

Supplement: Supplementary file 1 [file molecules-26-00361-s001.zip › images_for submission/DHE_Salsolinol/DHE_Salsolinol_original/K3G_DHE.tif]

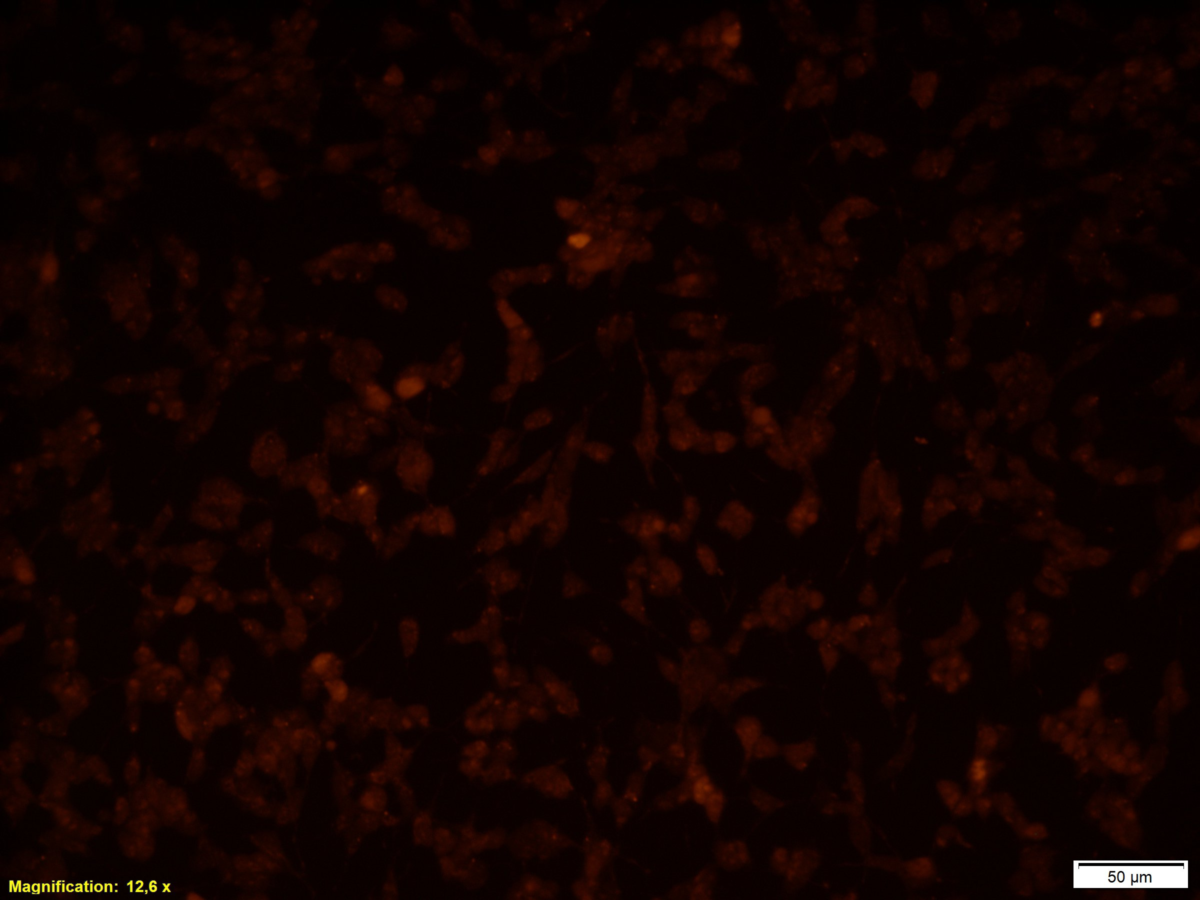

Supplement: Supplementary file 1 [file molecules-26-00361-s001.zip › images_for submission/DHE_Salsolinol/DHE_Salsolinol_original/NAC_DHE.tif]

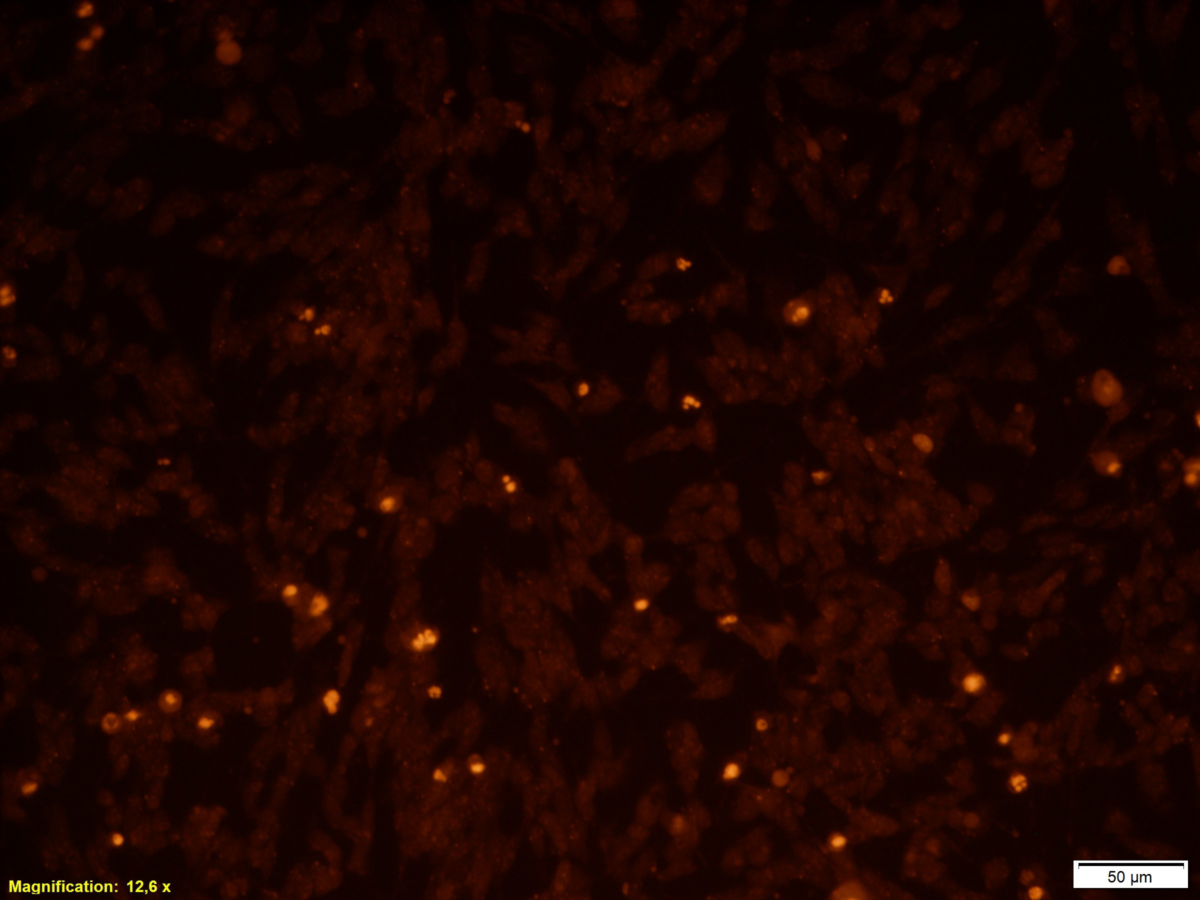

Supplement: Supplementary file 1 [file molecules-26-00361-s001.zip › images_for submission/DHE_Salsolinol/DHE_Salsolinol_original/SAL_DHE-tif.tif]

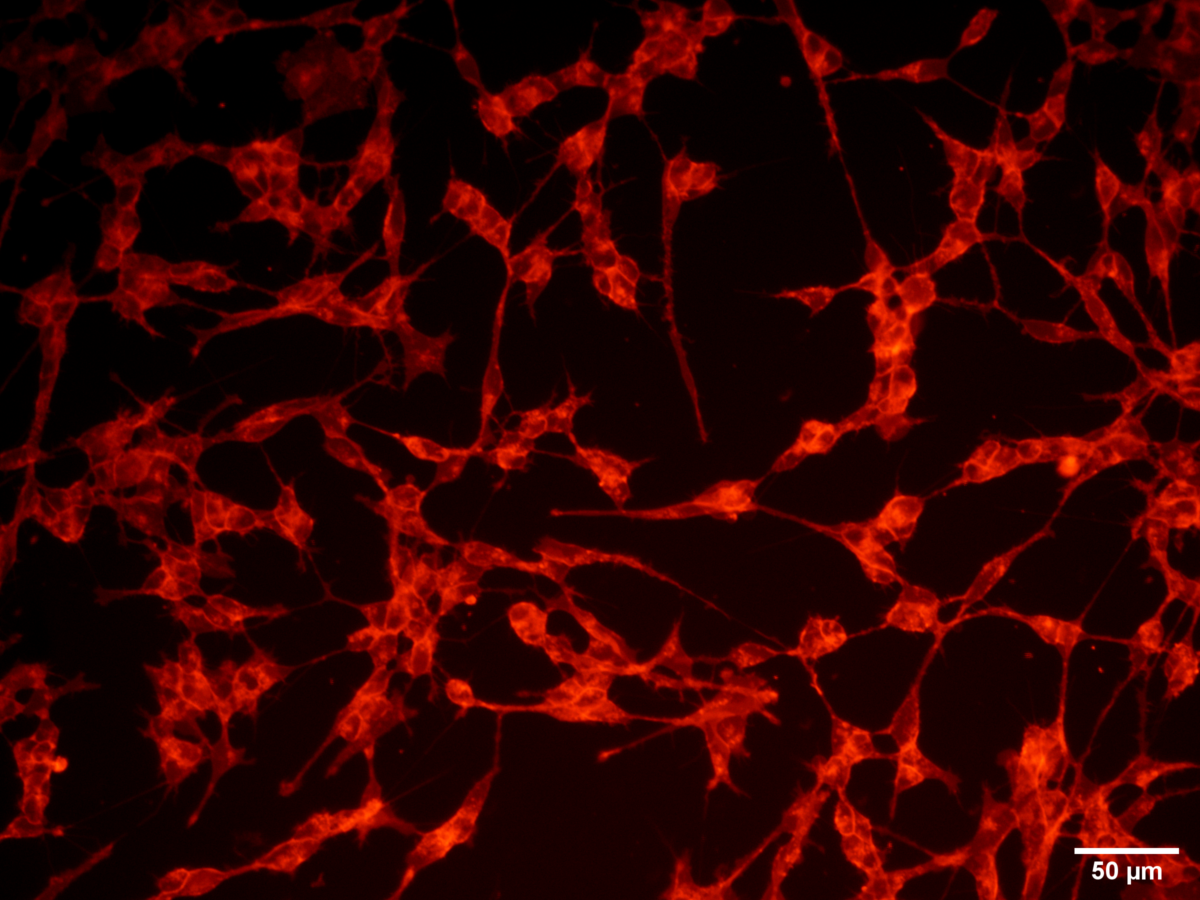

Supplement: Supplementary file 1 [file molecules-26-00361-s001.zip › images_for submission/differentiation/ATRA_processed.tif]

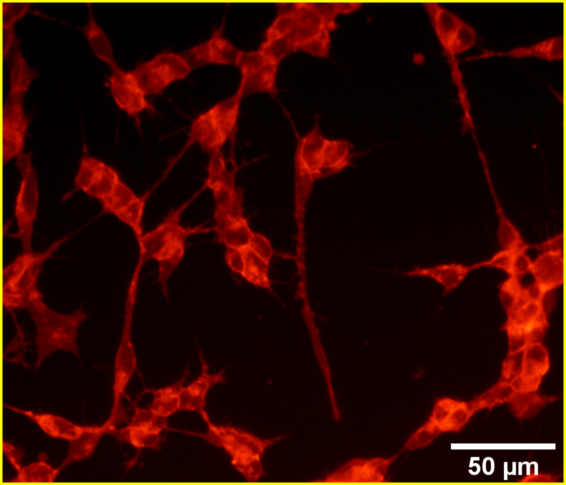

Supplement: Supplementary file 1 [file molecules-26-00361-s001.zip › images_for submission/differentiation/ATRA_processed_subarea.tif]

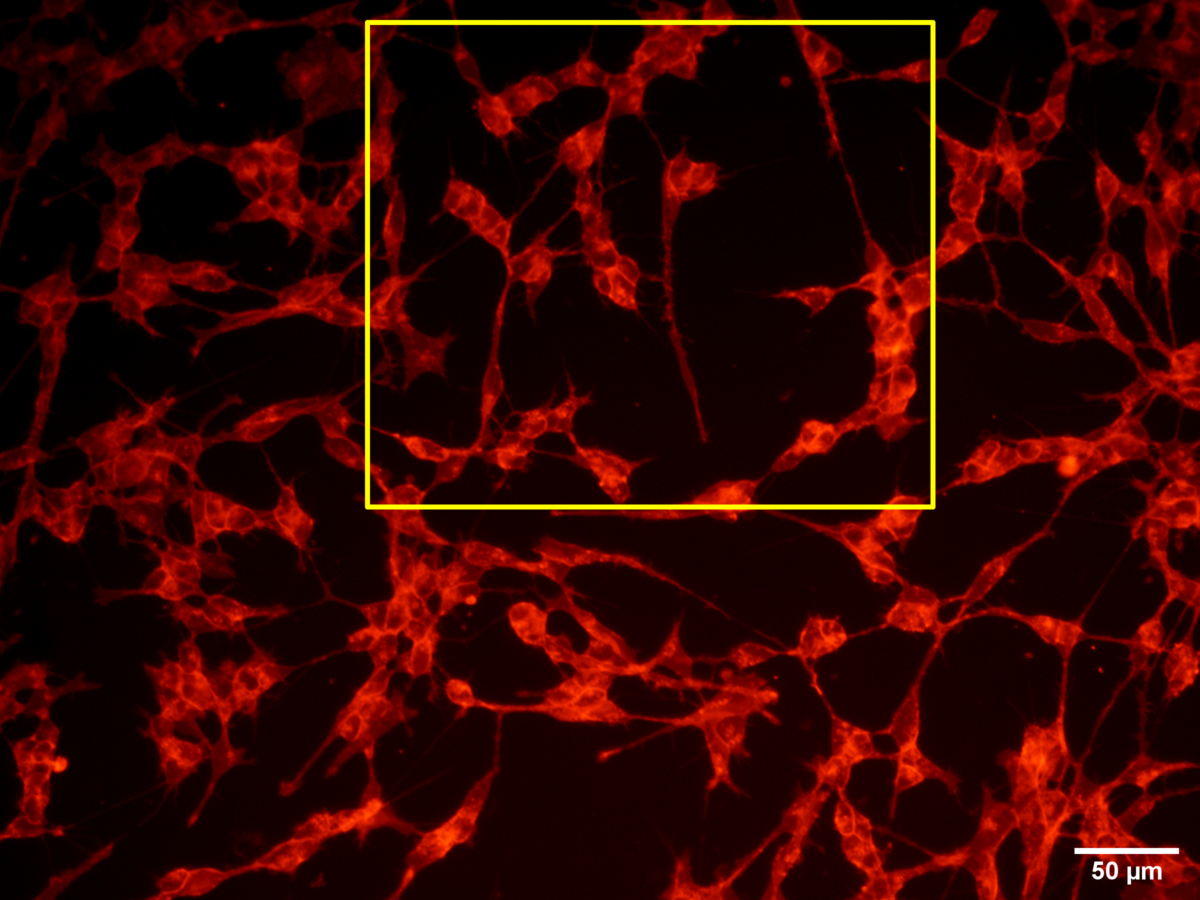

Supplement: Supplementary file 1 [file molecules-26-00361-s001.zip › images_for submission/differentiation/ATRA_processed_with_ROI.tif]

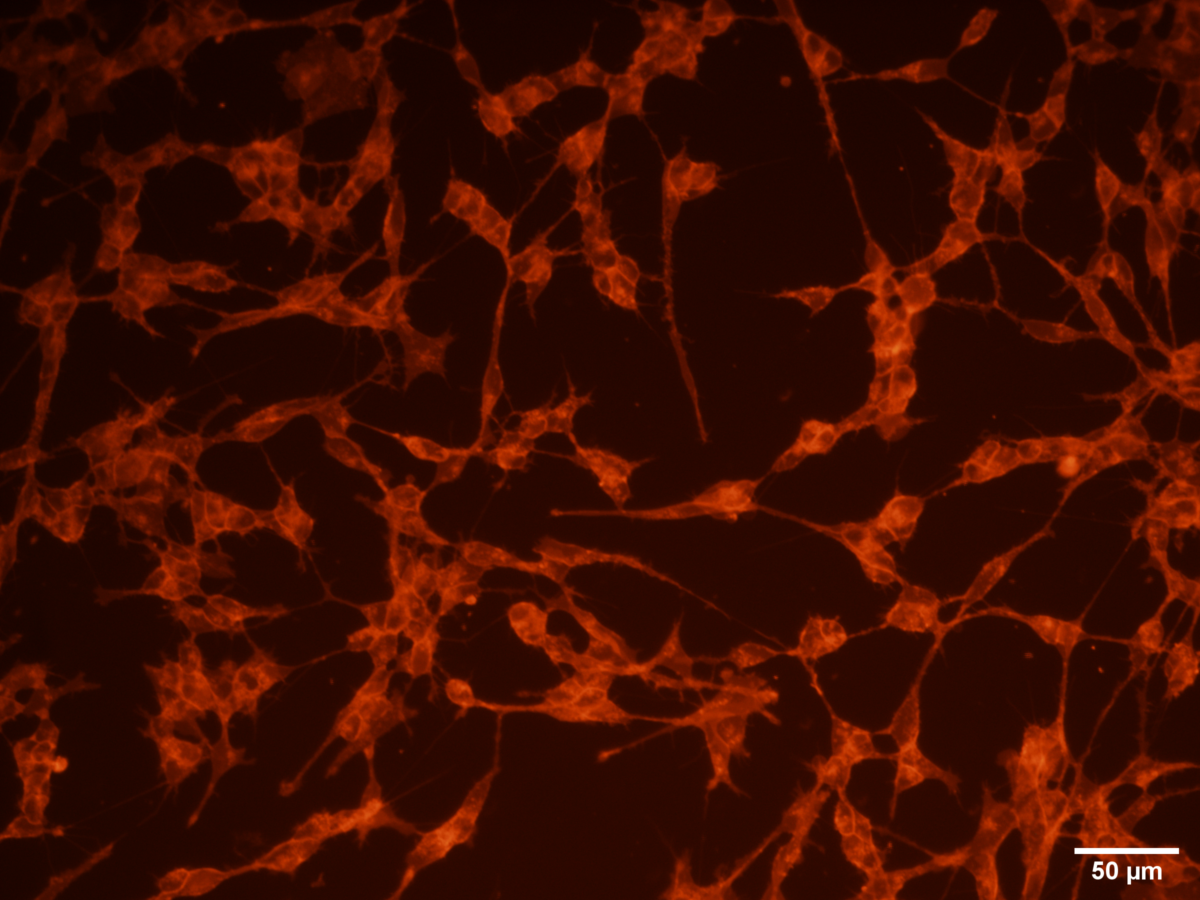

Supplement: Supplementary file 1 [file molecules-26-00361-s001.zip › images_for submission/differentiation/ATRA_raw.tif]

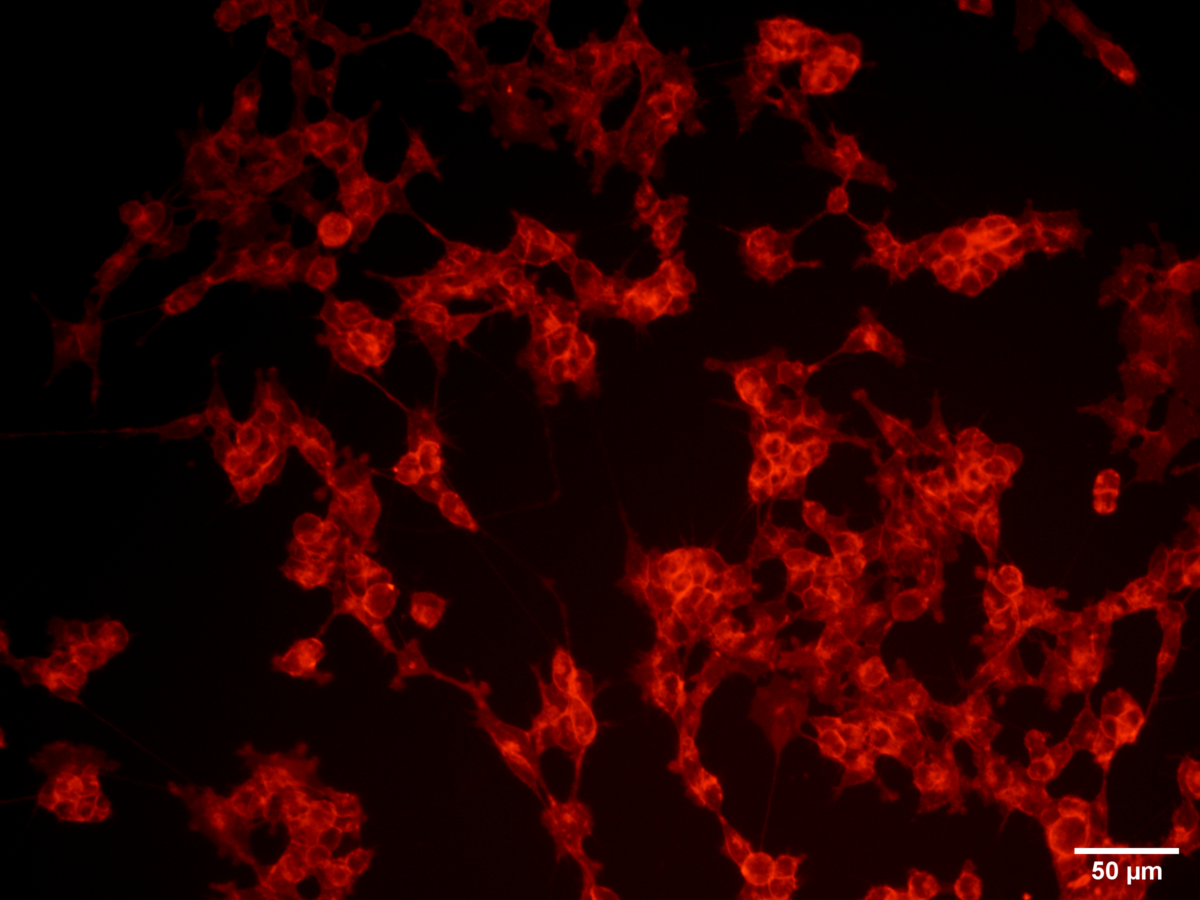

Supplement: Supplementary file 1 [file molecules-26-00361-s001.zip › images_for submission/differentiation/Undiff_processed.tif]

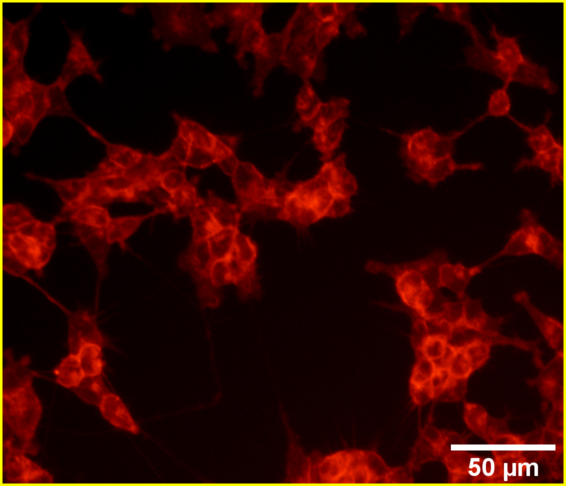

Supplement: Supplementary file 1 [file molecules-26-00361-s001.zip › images_for submission/differentiation/Undiff_processed_subarea.tif]

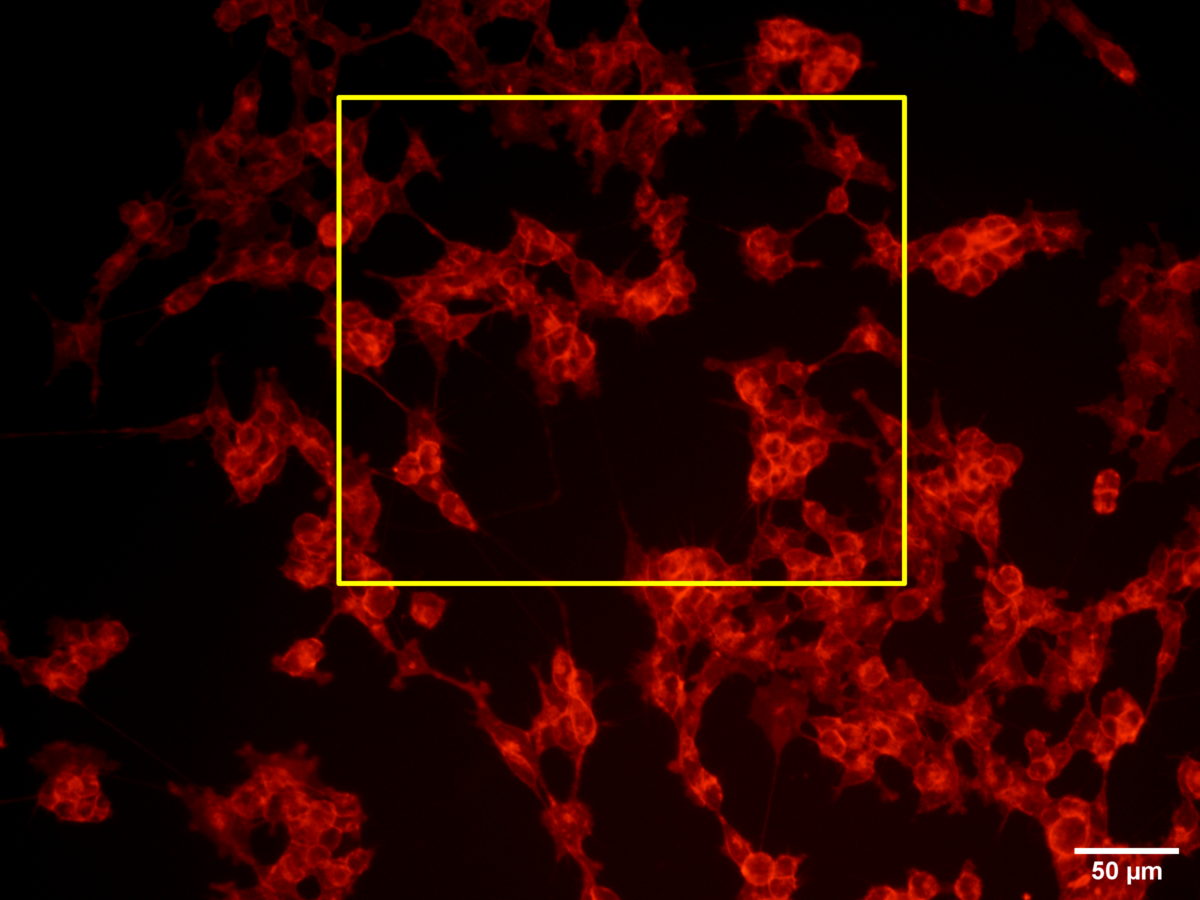

Supplement: Supplementary file 1 [file molecules-26-00361-s001.zip › images_for submission/differentiation/Undiff_processed_with_ROI.tif]

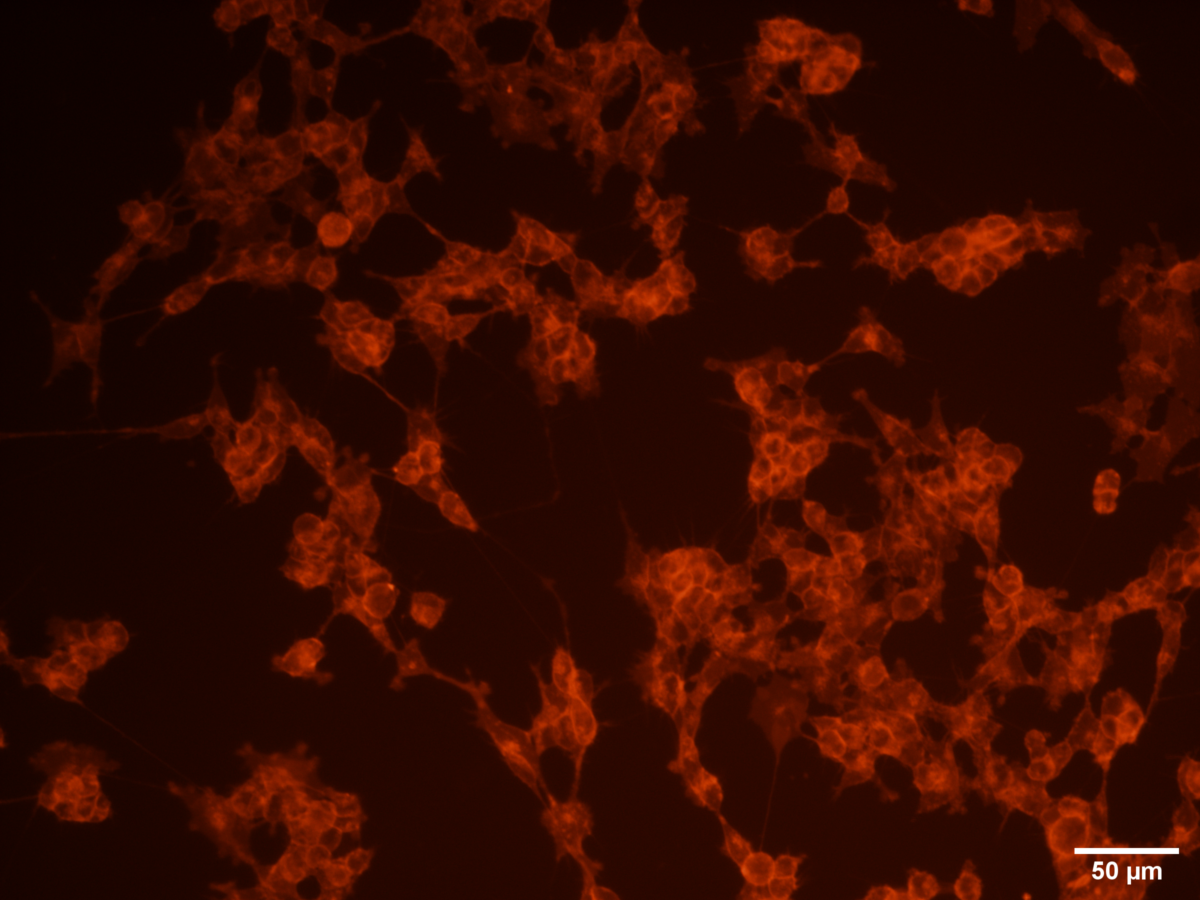

Supplement: Supplementary file 1 [file molecules-26-00361-s001.zip › images_for submission/differentiation/Undiff_raw.tif]
